# Arabidopsis SKP1-like protein13 (ASK13) positively regulates seed germination and seedling growth under abiotic stress

**DOI:** 10.1093/jxb/ery191

**Published:** 2018-05-18

**Authors:** Venkateswara Rao, Bhanu Prakash Petla, Pooja Verma, Prafull Salvi, Nitin Uttam Kamble, Shraboni Ghosh, Harmeet Kaur, Saurabh C Saxena, Manoj Majee

**Affiliations:** National Institute of Plant Genome Research (NIPGR), Aruna Asaf Ali Marg, New Delhi, India

**Keywords:** Abiotic stress, ASK13, F-box proteins, germination, proteasome, protein interaction, SCF complex, seedling growth, seed vigor

## Abstract

SKP1 (S-phase kinase-associated protein1) proteins are key members of the SCF (SKP–cullin–F-box protein) E3 ligase complexes that ubiquitinate target proteins and play diverse roles in plant biology. However, in comparison with other members of the SCF complex, knowledge of SKP1-like proteins is very limited in plants. In the present work, we report that Arabidopsis SKP1-like protein13 (ASK13) is differentially regulated in different organs during seed development and germination and is up-regulated in response to abiotic stress. Yeast two-hybrid library screening and subsequent assessment of *in vivo* interactions through bimolecular fluorescence complementation analysis revealed that ASK13 not only interacts with F-box proteins but also with other proteins that are not components of SCF complexes. Biochemical analysis demonstrated that ASK13 not only exists as a monomer but also as a homo-oligomer or heteromer with other ASK proteins. Functional analysis using *ASK13* overexpression and knockdown lines showed that ASK13 positively influences seed germination and seedling growth, particularly under abiotic stress. Taken together, our data strongly suggest that apart from participation to form SCF complexes, ASK13 interacts with several other proteins and is implicated in different cellular processes distinct from protein degradation.

## Teaser

In addition to participation in SCF complexes, Arabidopsis SKP1-like protein13, ASK13, positively influences seed germination and seedling growth, particularly under abiotic stress conditions.

## Introduction

Ubiquitin-mediated proteolysis by the 26S proteasome complex has been shown to be a central component in the regulation of several diverse biological processes in eukaryotes, mainly because of its ability to rapidly remove intracellular proteins (Hershko and Ciechanover, 1998; Moon *et al.*, 2004; Smalle and Vierstra, 2004; Dreher and Callis, 2007; Santner and Estelle, 2010). This ubiquitin-proteasome pathway exhibits more complexity in plant species than in animal systems and appears to control almost all physiological processes in plants (Smalle and Vierstra, 2004). The pathway employs several types of enzymes, with the ubiquitylation of the protein substrate being accomplished by the sequential action of three key enzymes: ubiquitin-activating enzyme (E1), ubiquitin-conjugating enzyme (E2), and ubiquitin protein ligase (E3). Subsequently, the resulting multi-ubiquitin-marked protein is recognized and degraded by the 26S proteasome complex (Hatfield *et al.*, 1997; Fu *et al.*, 1998; Hershko and Ciechanover, 1998; Pickart, 2001; Wolf and Hilt, 2004; Vierstra, 2009). Among the E3 ligase enzymes, the SCF (SKP–cullin–F-box protein) complex is the best understood and well characterized, despite being highly diverse. It consists of multi-protein complexes of SKP1 (S-phase kinase-associated protein1), CUL1/CDC53, RING finger protein RBX1/HRT/ROC1, and an F-box protein. Each component of this multi-subunit complex plays an important and distinct role. Cullin functions as a scaffold protein that interacts with SKP1 and RBX 1, while the SKP1-like protein serves as an adaptor that links CUL1/CDC53 and the F-box protein (Bai *et al.*, 1996; Weber *et al.*, 2005). Dysfunction of any of the core members of the complex causes severe defects in diverse developmental processes. Among the members of the SCF complexes, F-box proteins essentially impart substrate specificity to the complex and have therefore been subject to considerable research. Strikingly, about 700 F-box genes have been predicted in the *Arabidopsis thaliana* genome (Gagne *et al.*, 2002). Practically all hormone signaling pathways are controlled by F-box proteins, and even a few of the hormone receptors have been found to be F-box proteins (Dharmasiri *et al.*, 2005; Ueguchi-Tanaka *et al.*, 2005; Katsir *et al.*, 2008; Sheard *et al.*, 2010). It is notable that SKP1-like proteins in plants have been less studied compared to other members of the SCF complex. In contrast to a single *SKP1*-like gene present in yeast and humans (Bai *et al.*, 1996; Connelly and Hieter, 1996), SKP1-like proteins are encoded by a small gene family of 21 members in Arabidopsis (*Arabidopsis SKP1-like*, *ASK*) (Risseeuw *et al.*, 2003). Expression analyses revealed that *ASK* genes are differentially expressed in different organs (Zhao *et al.*, 2003; Takahashi *et al.*, 2004). Among the 21 ASK proteins, only ASK1 and ASK2 have been functionally characterized. Studies have shown that ASK1 and ASK2 exhibit overlapping functions that are essential for early patterning and development (Liu *et al.*, 2004). ASK1 has further been found to be involved in meiotic events and flower development (Yang *et al.*, 1999, 2006; Wang and Yang, 2006; Lu *et al.*, 2016). Recent studies have determined the roles of SKP proteins in abiotic stress tolerance in several plant species, including *Paeonia suffruticosa*, *Triticum aestivum*, and *Glycine soja* (Li *et al.*, 2012; Liu *et al.*, 2015; Hao *et al.*, 2017). Expression analysis of various ASK proteins in Arabidopsis has also suggested their participation in the seed germination processes (Marrocco *et al.*, 2003, Zhao *et al.*, 2003; Takahashi *et al.*, 2004; Dezfulian *et al.*, 2012; Kuroda *et al.*, 2012). The transition of seed from dormancy to germination is a complex physiological process that is highly influenced by both extrinsic and intrinsic factors. Furthermore, several positive regulators of seed dormancy act as negative regulators for seed germination (Bewley and Black, 1994; Bewley, 1997; Finch-Savage and Leubner-Metzger, 2006; Holdsworth *et al.*, 2008; Rajjou *et al.*, 2012). The proteasome pathway plays an important role in breaking dormancy as it has the ability to selectively dismantle the existing regulatory proteins and networks that repress the completion of seed germination and otherwise maintain seed dormancy. Phytochrome-interacting factor 1 (PIF1) and the DELLA group of proteins that act as negative regulators of seed germination are degraded through this proteasome pathway (Strader *et al.*, 2004; Oh *et al.*, 2006). The role of various F-box proteins that target these negative regulators has also been determined (Dill *et al.*, 2004; Strader *et al.*, 2004; McGinnis *et al.*, 2003). However, the role of SKP1-like proteins in seed germination has not been properly explored.

In addition to participation in SCF complexes, SKP1-like proteins form complexes with other proteins and are implicated in other cellular processes distinct from protein degradation. For example, SKP1 was shown to be an important component of the centromere-binding factor 3 (CBF3)–kinetochore complex that is required for the completion of the mitotic cell cycle (Connelly and Hieter, 1996). Furthermore, the single *SKP1* of *Saccharomyces cereviseiae* forms a complex with *Rav1* and *Rav2* termed RAVE, a regulator of (H^+^)-ATPase (V-ATPase) assembly (Seol *et al.*, 2001). However, in plants, apart from the involvement in SCF complexes, participation of SKP1-like proteins in other processes has not been properly examined. Therefore, the present study aimed to understand the role and regulation of SKP1-like proteins in seed germination and also to explore their participation in other processes besides protein ubiquitination.

In this work, we report that ASK13 (SKP1-like protein13; At3g60010) is differentially regulated in different organs during seed development and germination, and is up-regulated during abiotic stress. Through yeast two-hybrid library screening and bimolecular fluorescence complementation assays, ASK13 was found to interact with various F-box proteins and other proteins independent of SCF complexes. Molecular mass, subunit composition, and the possibility of homomeric and heteromeric associations among ASK proteins was also explored using gel filtration chromatography of the ASK13 protein overexpressed in bacteria and in Arabidopsis. Finally, the role of ASK13 in seed germination and seedling growth was addressed through the generation and functional characterization of *ASK13*-overexpression and *ask13*-RNAi-lines.

## Materials and methods

### Plant material and growth conditions

*Arabidopsis thaliana* (Col-0) seeds were used for all the experiments in the present study. All plants were grown in growth chambers under controlled conditions of 22 ± 2 °C and a 16/8 h light/dark cycle with light intensity 100 μm m^–2^ s^–1^).

### Isolation and molecular cloning of *ASK13* (*At3g60010*)

Total RNA was isolated from Arabidopsis seeds according to Singh *et al.* (2003) and then cDNA was prepared using a cDNA synthesis kit (Applied Biosystems). Full-length cDNA of *ASK13* was amplified using gene-specific primers (Supplementary Table S1 at *JXB* online), which were designed based on available gene sequence data in The Arabidopsis Information Resource (TAIR, http://arabidopsis.org). The amplicon was cloned in the pJET1.2 vector (Fermentas) and subsequently sequenced.

### RNA extraction and quantitative real-time PCR

Total RNA was isolated from individual Arabidopsis organs and seedlings using TRI Reagent (Sigma). For seeds and siliques, RNA was extracted according to Singh *et al.*, (2003). Total RNA (2 μg) treated with DNase I was reverse-transcribed using random primers (cDNA synthesis kit, Applied Biosystems). Real-time PCR reactions were run on a thermocycler (StepOne Real-Time PCR System, Applied Biosystems) using gene-specific primers for *ASK13* and an endogenous control for the ribosomal *18S* subunit (Supplementary Table S1). The expression of *ASK13* mRNA was normalized to the expression of *18S* mRNA. For each real-time PCR reaction, a 20-μl mixture containing 2 μl of diluted (1:20) cDNA, 10 μl of power SYBR green PCR master mix (Applied Biosystems), and 20 pmol of each forward and reverse primer were used. A negative control lacking the RT enzyme was also included in each experiment. All reactions were performed in triplicate, with at least three biological replicates.

### Yeast two-hybrid assay (Y2H)

For Y2H library screening, *ASK13* was initially sub-cloned into the bait vector pGBKT7BD and transformed into the Y2H Gold strain (Clonetech) using an EZ-Yeast Transformation Kit (MP Biomedicals) according to the manufacturers’ instructions. The pGBKT7BD-*ASK13* transformed cells were then mated with a normalized Arabidopsis Y2H cDNA library (purchased from Clonetech) and screening was carried out according to the manufacturer’s guidelines. Selection was performed on SD plates lacking histidine, tryptophan, leucine, and adenine but supplemented with X-α-Gal and aureobasidin A. For Y2H one-to-one interactions, the Gateway-compatible vectors pDEST-GBKT7 and pDEST-GADT7 (from TAIR) were used. Selected F-box, non-F-box, and several other ASK cDNAs were cloned into the pENTR/D-TOPO vector using Gateway cloning technology (Invitrogen) and eventually cloned in the pDEST-GADT7 Y2H vector. The resulting plasmids were co-transformed to the Y2H gold strain and then the transformed cells were selected on SD–Ade–His–Leu–Trp/X-α-Gal/aureobasidin agar media. pGADT7-T- and pGBKT7-Lam-transformed Y2H gold strain was used as a negative control and pGADT7-T- and pGBKT7-53-transformed Y2H gold strain was used as a positive control for interactions.

### Bacterial overexpression and purification of recombinant *ASK13*

*ASK13* was sub-cloned into the bacterial expression vector pET23d (Novagen) to express as a C-terminal histidine-tag fusion protein in *E. coli* BL-21(DE3) cells. Transformed *E. coli* cells were grown in LB broth at 37 °C until they attained an absorption of 0.5 units at 600 nm (A_600_) of 0.5 and they were then induced by 0.5 mM IPTG for 8 h at 25 °C. Proteins expressed in the soluble fractions were purified to homogeneity using nickel charged affinity columns (GE Healthcare).

### SDS and native PAGE analysis

SDS PAGE was performed according to the protocol described by Laemmli (1970) and for native PAGE analysis all components except SDS and β-mercaptoethanol were used. Native-PAGE gels (10%) were run at 200 V for ~1 h in a BioRad Mini-PROTEAN gel system according to the manufacturer’s protocol. The gel was stained by CBB-R250 in staining solution followed by destaining to visualize the protein bands.

### Protein identification

Protein identification and peptide analysis was carried out using a 4800 MALDI-TOF/TOF (Applied Biosystems/MDS SCIEX) at the National Institute of Plant Genome Research, India, proteomics facility as described in Kaur *et al.* (2015).

### Protein extraction from plants

Protein from green fluorescent protein (GFP)-fused ASK13 transgenic Arabidopsis seedlings was isolated according to Verma *et al.* (2013). Tissue was ground to a fine powder in liquid nitrogen using a mortar and pestle, and 1 ml of protein extraction buffer was added [100 mM HEPES buffer, pH 7.5, 1 mM β-mercaptoethanol, and protease inhibitor cocktail (Sigma)]. The suspension was centrifuged at 13000 *g* for 15 min at 4 °C, the supernatant was collected, and the protein content was estimated using the Bradford protein assay (Bradford, 1976).

### Size-exclusion chromatography and dot blot analysis

The molecular mass of GFP-fused ASK13 protein was determined by size-exclusion chromatography according to Petla *et al.* (2016). Gel filtration was carried out on an AKTA Basic FPLC system (GE Healthcare) using a Sephacryl 200HR pre-packed column (GE Healthcare). To generate standard curves, 12.4-kDa cytochrome C, 29-kDa carbonic anhydrase, 66-kDa BSA, and 150-kDa alcohol dehydrogenase (Sigma) were run on the column and the elution volume, *V*_e_, was determined; 2000-kDa Blue Dextran was used to determine the void volume, *V*_o_. A standard graph was plotted with *V*_e_/*V*_o_ on the *x*-axis and molecular mass on the *y*-axis on a logarithmic scale. Protein samples (2 mg) were loaded and run at a constant flow rate of 0.4 ml min^–1^ and 1-ml fractions were collected. Fractions were loaded on a polyvinylidene difluoride (PVDF) membrane using a dot-blot apparatus, and the dot blot was performed according to Petla *et al.* (2016).

### Bimolecular fluorescence complementation (BiFC) assays

*ASK13* was cloned in the pENTR/D-TOPO vector and transferred to the pSAT4-DEST-N (1–174) EYFP-C1 and pSAT5-DEST-C (175-END) EYFP-C1 vectors. The F-box genes *At1g08710* and *At5g45360* and the non-F-box genes *At4g32530* and *At2g23070* were cloned to the pENTR/D-TOPO vector following amplification using the appropriate primer set (Supplementary Table S1) and were sub-cloned into the pSAT5-DEST-C (175-END) EYFP-C1 vector using Gateway cloning technology (Invitrogen). The plasmid DNAs of respective constructs were bombarded into onion (*Allium cepa*) epidermal cells using a helium-driven gene gun system (PDS-1000; Bio-Rad, USA) according to the manufacturer’s instructions. For the negative control, pSAT4-DEST-N (1–174 N-terminus half of yellow fluorescent protein, YFP) EYFP-C1, and pSAT5-DEST-C (175-END C-terminus half of YFP) empty vectors were bombarded into onion epidermal cells. The onion peels were kept on Murashige and Skoog (MS) plates in darkness for 24 h. A True Confocal Scanner (TCS) Superior image Performance version 2 (SP2) Acousto-Optical Beam Splitter (AOBS) laser confocal scanning microscope (Leica Microsystems) was used to study the interaction (YFP signal).

### Plasmid construction and generation of transgenic plants

To make the *ASK13* overexpression construct, cDNA of full-length *ASK13* was amplified using gene-specific primers (Supplementary Table S1) and cloned into the pJET1.2 vector. *ASK13* was subsequently ligated downstream of the CaMV35S promoter of the binary vector pCAMBIA2301. For the *ASK13::GFP* fusion construct, *ASK13* was initially cloned into the pKYLX80:GFP vector. Finally, *ASK13* fused with *GFP* from the *pKYLX80::ASK13-GFP* construct was transferred to the binary vector pCAMBIA2301. For intron-spliced hairpin RNAi lines, unique sequences of *ASK13* cDNA were chosen as the targeted interference region. The unique sequences of the *ASK13* region together with the 3′-UTR were amplified using sequence-specific primers (Supplementary Table S1) and were initially cloned into the pENTR/D-TOPO vector, and then transferred to the Gateway-compatible RNAi vector pHELLSGATE 12. For promoter analysis, a construct was made of the *ASK13* promoter fused with the β-glucuronidase (GUS) reporter gene. These constructs were transformed to Arabidopsis plants using the floral dip method (Clough and Bent, 1998) through *Agrobacterium*-mediated transformation. For complementation studies, *ask13* mutant plants were used for transformation. Transgenic plants were selected based on suitable antibiotic resistance followed by transcript analysis.

### GUS staining

Seedlings, leaves, flowers, siliques, and seeds without the seed coat were incubated overnight in a GUS staining solution [100 mM phosphate buffer (pH 7.0), 1 mM X-gluc (Gold biotechnology, St. Louis, MO), 10 mM EDTA, 0.1 % (v/v) Triton X-100] at 37 °C. Tissue samples were then treated with 95% (v/v) ethanol to remove chlorophyll and photographed under a microscope (Nikon Stereo Zoom).

### Subcellular localization of *ASK13*

Subcellular localization of *ASK13* was determined in 5-d-old *GFP*-fused *ASK13* transgenic seedlings. Roots of the transgenic seedling were observed using a TCS SP2 (AOBS) laser confocal scanning microscope (Leica Microsystems) for GFP fluorescence.

### Analysis of mutant seeds

Mutant seeds of *ask13* (CS466214) were obtained from the GABI-Kat seed collection (https://www.gabi-kat.de/). Homozygous lines were selected by PCR using the T-DNA left border and gene-specific primers (Supplementary Table S1) with extracted genomic DNA from mutant plants. DNA bands obtained in homozygous plants were excised from agarose gels and sequenced to identify the site of insertion. T-DNA was found to be inserted in the 3′-UTR region (Supplementary Fig. S9). Final confirmation of the mutant seeds was performed by transcript analysis.

### Seed germination assay

All transgenic, mutant, and wild-type plants were grown under identical controlled conditions in a growth chamber (22 ± 2 °C, 16/8 h light/dark cycle with light intensity 100 μm m^–2^ s^–1^). Seeds were harvested on the same day and then stored in the dark under dry conditions at room temperature (24 ± 2 °C) for at least 8 weeks before use in a germination assay. Seeds were harvested from at least four different plants of each type. Four biological replicates of 50 seeds of each were used for analysis. Germination assays were performed according to Saxena *et al.* (2013). Seeds were surface-sterilized with 30% (v/v) sodium hypochlorite solution and then washed several times with MilliQ water. For stratification, all seeds were kept moist in darkness at 4 °C for 3 d. To assess germination efficiency under stress conditions, batches of seeds were then plated on half-strength MS agar medium supplemented with either sodium chloride (150 mM), polyethylene glycol (PEG 4000; –0.4 MPa), or paraquat (PQ; 0.3 μM) and transferred to the growth chamber. For cold stress, plates (half-strength MS medium) containing seeds were kept at 4 °C. For heat stress, seeds were treated at 45 °C for 2 h before being plated on half-strength MS medium and transferred to the growth chamber. Germination was scored after 7 d for all treatments. Seeds were considered to have completed germination when the radicle protruded from the testa.

### Seedling growth, stress treatments, and phytohormone treatments

All the stress treatments were performed according to Saxena *et al.* (2013). Wild-type, *ASK13* overexpression, and mutant seeds were germinated on half-strength MS agar medium in a growth chamber at 24 ± 2 °C, 16/8 h light/dark cycle with light intensity 100 μm m^–2^ s^–1^. For all the experiments 7-d-old seedlings were used. Cold stress was applied at 4 °C and heat stress was applied at 37 °C. For salt stress 150 mM NaCl was added to the medium, for oxidative stress 2 μM PQ was added, and for osmotic stress PEG (–0.5 MPa) was added. For hormone treatments, the 7-d-old seedlings in the growth chamber were placed for 6 h in hormone solutions in Petri dishes containing either 10 µM auxin (indole-3-acetic acid, IAA), abscisic acid (ABA), gibberellic acid (GA), salicylic acid (SA), methyl jasmonate (JA), ethylene (1-aminocyclopropane-1-carboxylic acid, ACC), or cytokinin (6-benzylaminopurine, BAP).

### Controlled deterioration treatment (CDT)

All the seeds that used in this experiment were harvested on the same day from dry siliques. The CDT was performed according to Salvi *et al.* (2016). Before exposing seeds to higher temperatures, the moisture content was increased to 22 ± 2% by imbibing seeds in water for 1 h followed by air drying. Seeds were incubated at 45 °C (0–4 d) at 70% relative humidity and then used for evaluation of viability and germination percentages. Experiments were carried out in triplicate using 50 seeds in each batch.

### Tetrazolium assay

A tetrazolium (TZ, 2,3,5-triphenyl tetrazolium chloride) assay was performed according to Verma and Majee (2013). Seeds were scarified using sodium hypochlorite solution (30% v/v), washed several times with MilliQ water, and then treated with 1% TZ solution at 30 °C for 48 h. The viability of the seeds was determined according to the staining pattern and color intensity.

### Accumulation of reactive oxygen species (ROS) and malondialdehyde (MDA)

Seedlings were grown to 7 d old in a growth chamber at 24 ± 2 °C, 16/8 h light/dark cycle with light intensity 100 μm m^–2^ s^–1^. The H_2_O_2_ content of seedlings was determined according to the method described by Alexieva *et al.* (2001). A sample of tissue (0.2 g) was homogenized in 2 ml of 0.1% (w/v) trichloroacetic acid (TCA) and the supernatant was collected after centrifugation at 13000 *g* for 20 min at 4 °C. The reaction mixture was prepared with 0.5 ml of supernatant, 1 ml of 1 M potassium iodide, and 0.5 ml of 10 mM potassium phosphate buffer and incubated in darkness for 1 h. Absorbance was measured at 390 nm. The H_2_O_2_ content was calculated by using a standard curve generated with known concentrations of H_2_O_2_.

The MDA content was measured according to Heath and Packer (1968). A sample of tissue (200 mg) was homogenized in 2 ml of 0.25% 2-thiobarbituric acid (TBA), which was prepared in 10% TCA and incubated at 95 °C for 30 min. This reaction mixture was kept on ice for 10 min. The supernatant was collected after centrifugation at 13000 *g* for 30 min. Absorbance of the supernatant was measured at 532 and 600 nm. The concentration of the MDA was calculated using an extinction coefficient of 155 mM^−1^ cm^−1^.

### Statistical analysis

Differences in percentage germination at representative time-points after imbibition, H_2_O_2_ and MDA contents, and transcript abundances among genotypes were all assessed by one-way ANOVA using the SPSS program (SPSS, Chicago, IL). All data were subjected to ANOVA and if this indicated that there were significant differences among means, Duncan’s Multiple Range Test was used to distinguish among them at α=0.01.

## Results

### *ASK13* is highly expressed in developing seeds and is up-regulated under abiotic stress

**Fig. 1.**
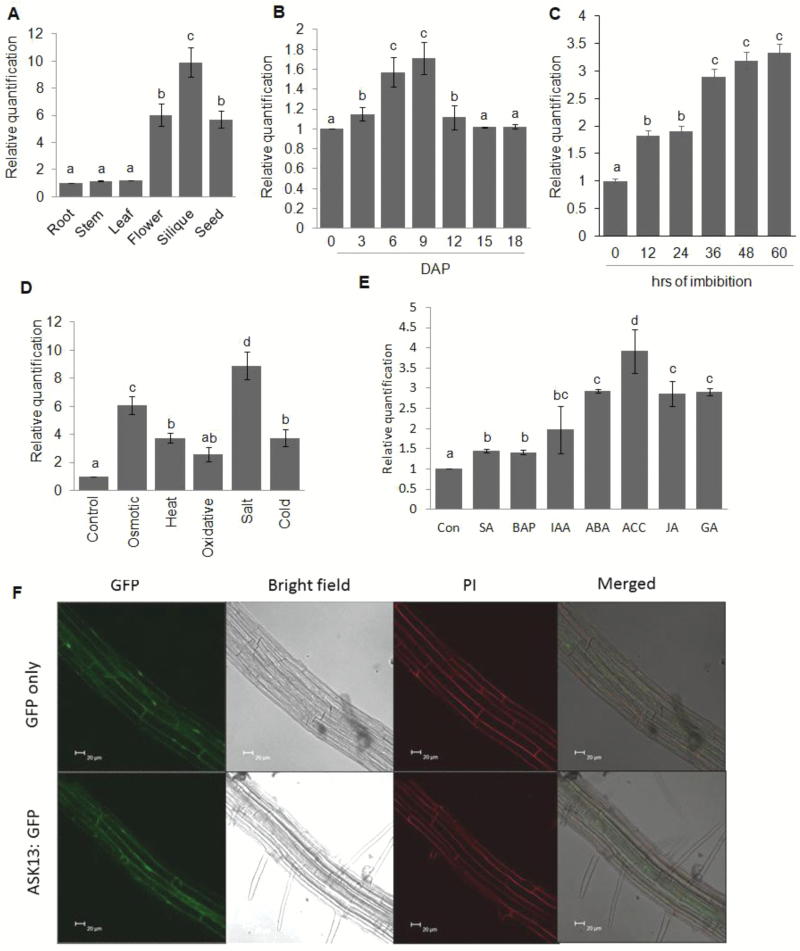
Quantitative RT-PCR analysis of *ASK13* (*At3g60010*) in (A) leaves, roots, stem, and flowers of 6-week-old mature plants, siliques with seeds at 14–16 d after pollination (DAP), and seeds after-ripened for 8 weeks; (B) during seed development; (C) during seed germination; (D) in 7-d-old seedlings exposed to various forms of abiotic stress; and (E) in 7-d-old seedlings treated with various phytohormones (Con, control; SA, salicylic acid; BAP, 6-benzylaminopurine; IAA, indole-3-acetic acid; ABA, abscisic acid; ACC, 1-aminocyclopropane-1-carboxylic acid; JA, methyl jasmonate; GA, gibberellic acid). Total RNA from each sample was reverse-transcribed and subjected to real-time PCR analysis. The relative expression value of each gene was normalized using *18S* ribosomal small subunit RNA as the endogenous control, and calculated using the 2^–ΔΔ*C*T^ method (Livak and Schmittgen, 2001). Data are means (±SD) from triplicate analyses of three biological replicates. Different letters indicate significant differences according to Duncan’s Multiple Range Test (*P*<0.01). (F) Subcellular localization of ASK13 as determined using green fluorescent protein (GFP). Images show localization of GFP and ASK13-GFP in roots of 5-d-old transgenic Arabidopsis seedlings. Cells were stained with propidium iodide (PI), and the emission signal was detected between 580–680 nm. Bright-field and merged images are also shown.

To begin to understand the role and the protein-binding repertoire of *ASK13*, we initially analysed the accumulation of transcripts in different organs in Arabidopsis. The results showed that *ASK13* was predominantly expressed in reproductive organs, with maximum abundance in siliques followed by seeds and flowers (Fig. 1A). Low levels of transcripts were also observed in the vegetative organs. The promoter-GUS analysis also confirmed the expression of *ASK13* in these tissues (Supplementary Fig. S1A). To obtain a more detailed picture of transcript accumulation over time, flowers were tagged the day after pollination and the subsequent transcript accumulation of *ASK13* was analysed throughout seed development. Accumulation was found to increase during the early phase of seed development until 9 d after pollination (DAP) and then it declined slightly towards seed maturation (Fig. 1B). Transcript accumulation then progressively increased as seed germination proceeded and reached maximum at 60 h of imbibition when seeds completed 100% germination (Fig. 1C). Transcript accumulation was also analysed in seedlings exposed to various forms of abiotic stress and transcript was enhanced to varying degrees (Fig. 1D), With maximum levels of induction being observed under salinity and osmotic stress. *ASK13* transcript accumulation was also found to be enhanced to varying degrees by all the phytohormones that were tested (Fig. 1E).

Subcellular localization of ASK13 was determined through confocal visualization of the stably expressed ASK13-GFP-fused protein. *ASK13* was expressed in a constitutive manner as the C-terminal GFP fused protein (ASK13-GFP). Examination of roots of 5-d-old transgenic seedlings demonstrated that ASK13 expression occurred throughout the cell including the nucleus and cytoplasm (Fig. 1F, Supplementary Fig. S1B). ASK protein members have previously been shown to be localized in both the nucleus and cytoplasm (Dezfulian *et al.*, 2012).

### ASK13 interacts with F-box proteins and several other proteins independent of SCF complexes

**Fig. 2.**
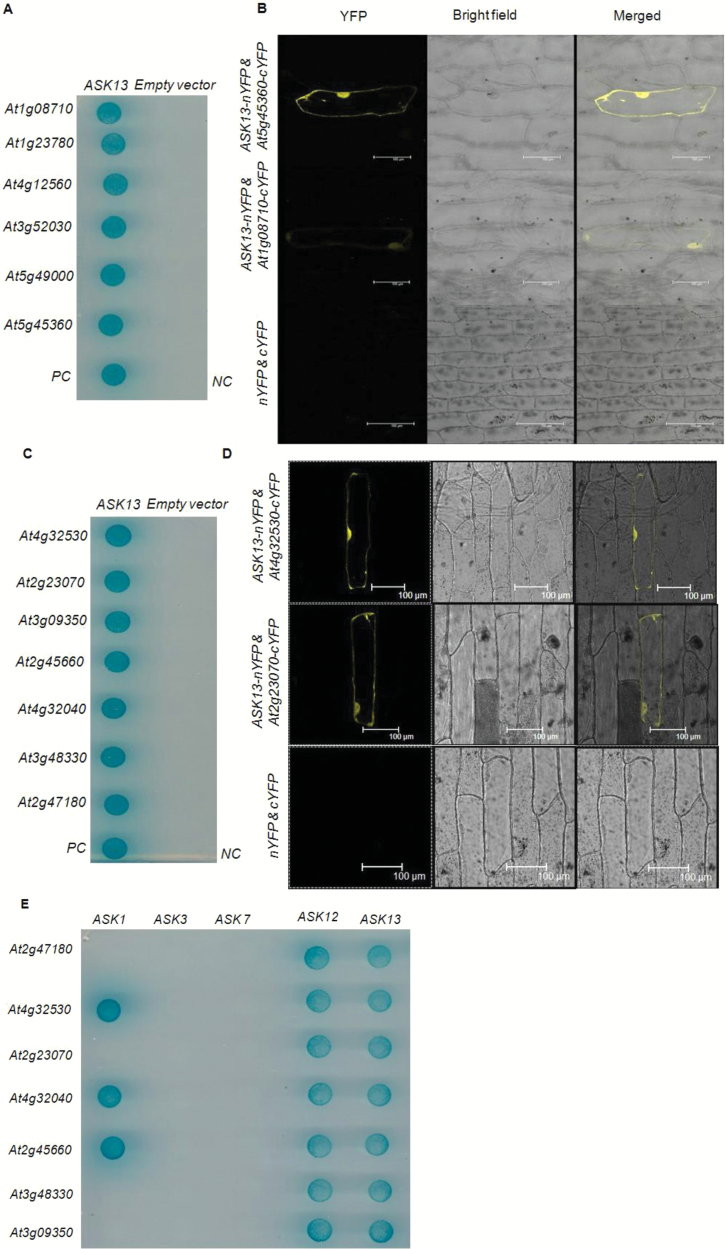
Interaction analysis of ASK13 using yeast two-hybrid (Y2H) (A, C, E) and bimolecular fluorescence complementation (BiFC) (B, D) assays. (A, C) Y2H interactions among ASK13 and selected F-box (A) and non-F-box (C) proteins. Yeast cells were co-transformed with pDEST-GBKT7::*ASK13* and grown on yeast synthetic drop-out medium lacking leucine, tryptophan, histidine, and adenine supplemented with X-α-Gal and aureobasidin A. Auto-activation of the proteins was analysed by co-transforming them with the pDEST-GBKT7 empty vector. PC, positive control; NC, negative control for interaction. (B, D) Interaction analysis of ASK13 with (B) F-box proteins (At5g45360 and At1g08710) and (D) non-F-box proteins (At4g32530 and At2g23070) using BiFC assays in onion epidermal cells. The bottom panel shows the negative control, where empty BiFC vectors (cYFP + nYFP) were co-transfected. (E) Y2H analysis of ASK proteins and non-F-box proteins. Yeast cells were co-transformed with pDEST-GBKT7 *ASK* cDNAs and pDEST-GADT7 non-F-box cDNAs indicated and were grown on synthetic drop-out medium lacking leucine, tryptophan, histidine, and adenine supplemented with X-α-Gal, aureobasidin A.

In addition to interactions with F-box and cullin proteins and consequent involvement in SCF complexes, SKP1-like proteins have also been shown to interact with other proteins and to participate in other cellular processes that are independent of SCF complexes in animal systems (Connelly and Hieter, 1996; Seol *et al.*, 2001). However, such interactions of SKP1-like proteins with other proteins have not been properly explored in plant systems. We therefore initially attempted to identify the interacting proteins of ASK13 through Y2H library screening. A normalized Arabidopsis Y2H cDNA library was chosen as prey and screened with ASK13 (bait). The Y2H library was constructed from mRNA isolated from 11 Arabidopsis tissues, namely seedlings (four developmental stages), etiolated seedlings, open flowers, buds, pollen, siliques, leaves, and stems. To ensure that ASK13 alone was not able to activate the yeast reporter genes, the *ASK13* bait construct was first evaluated for self-activation before further screening proceeded. Initial positive interactions were repeated by screening and eventually more than 70 potential interactive colonies were identified. Plasmids were retrieved from interacting colonies and each was tested for auto-activation with an empty bait vector control and also for interactions with ASK13. These screening processes identified 36 potential interactors. Sequencing these plasmids identified a wide range of proteins belonging to the F-box protein family and several non-F-box proteins, and also ASK13 itself (Table 1). To gain further insights, one-to-one interactions between ASK13 and a few randomly selected F-box- and non-F-box- proteins were examined through Y2H analyses. Full-length cDNAs of the proteins from the total cDNAs were amplified, independently cloned in the pDEST-GADT7 vector, and one-to-one Y2H analysis was then carried out. The results again confirmed that *ASK13* interacted proteins interacted not only with F-box proteins but also with other proteins that are not members of SCF complexes (Fig. 2A, C). *In vivo* interactions between ASK13 and non-F-box proteins were further confirmed by BiFC analysis. *ASK13* was transiently expressed as a YFP N-terminal fusion protein and selected F-box- and non-F-box- proteins were transiently expressed as YFP C-terminal fused proteins in onion epidermal cells. The BiFC results showed that ASK13 interacted with both the F-box and the non-F-box proteins identified using Y2H (Fig. 2B, D). Next, we examined interactions between the non-F-box proteins and several other SKP1-like proteins. The Y2H analyses revealed that such interactions did occur, and thus interactions were not limited to the ASK13 protein alone (Fig. 2E). Expression profiles of coding genes for ASK13-interacting proteins were analysed using affymetrix expression microarrays and Genevestigator (Hruz *et al.*, 2008), and several F-box and non-F-box proteins were found to be highly expressed in seeds (Supplementary Fig. S2).

**Table 1.** List of ASK13-interacting proteins and their functions

Serial no.	Interactive protein gene ID	Interacting protein description	Gene ontology
F-box proteins			
1	*At1g08710*	F-box family protein	Unknown
2	*At1g23780*	F-box family protein	Unknown
3	*At3g52030*	F-box family protein	Unknown
4	*At5g49000*	F-box family protein	Unknown
5	*At5g45360*	F-box family protein	Unknown
6	*At4g12560*	CPR1/CPR30 (F-box protein)	Pathogenesis
Non-F-box proteins			
7	*At4g32530*	ATPase, F0/V0 complex, subunit C protein	Germination
8	*At2g23070*	Protein kinase superfamily protein	Abiotic stress
9	*At3g09350*	Hsp70-binding protein1	Abiotic stress
10	*At2g45660*	Agamous-like 20	Germination, flower development
11	*At4g32040*	Knotted1-like homeobox gene 5	Root development
12	*At3g48330*	Protein isoaspartyl methyl transferase 1	Seed vigor and stress
13	*At2g47180*	Galactinol synthase 1	Abiotic stress
14	*At2g47210*	Myb-like transcription factor family protein	Unknown
15	*At2g41100*	Calmodulin-like 4	Protein phosphorylation
16	*At3g27850*	Ribosomal protein L12-C	Unknown
17	*At5g54810*	Tryptophan biosynthesis 2	Abiotic stress
18	*At1g56090*	Tetratricopeptide repeat (TPR)-like superfamily protein	Unknown
19	*At5g55300*	DNA Topoisomerase 1 alpha subunit	Morphogenesis
20	*At4g17730*	Syntaxin of plants 23	Unknown
21	*At1g51690*	Regulatory subunit of protein phosphatase 2A	Regulation protein phosphatase 2A
22	*At4g12340*	Copper ion binding protein	Unknown
23	*At3g16500*	Indole-3-acetic acid inducible 26	Regulates PHYA-induced CHS expression through HY5
24	*At5g19820*	Embryo defective 2734	Unknown
25	*At1g16870*	Mitochondrial 28S ribosomal protein	Unknown
26	*At4g34920*	PLC-like phosphodiesterases superfamily protein	Unknown
27	*At1g12250*	Pentapeptide repeat containing protein	Unknown
28	*At1g15700*	Chloroplast ATP synthase gamma subunit	Light signaling
29	*At3g04700*	DUF1685	Unknown
30	*At2g41440*	Unknown protein	Unknown
31	*At5g36700*	2-Phosphoglycolate phosphatase 1	Photorespiration
32	*At1g02280*	ATTOC33, Plastid protein import 1	Required for import of POR B into chloroplasts
33	*At2g04700*	Ferredoxin thioredoxin reductase	Unknown
34	*At3g60010*	Arabidopsis SKP1-like protein13	Unknown
35	*At2g47590*	Photolyase/blue light receptor	Blue-light signaling
36	*At4g20020*	Multiple organellar RNA editing factor 1	RNA editing in mitochondria

### ASK13 exists in monomeric, homo-oligomeric, and heteromeric associations with other ASK proteins

**Fig. 3.**
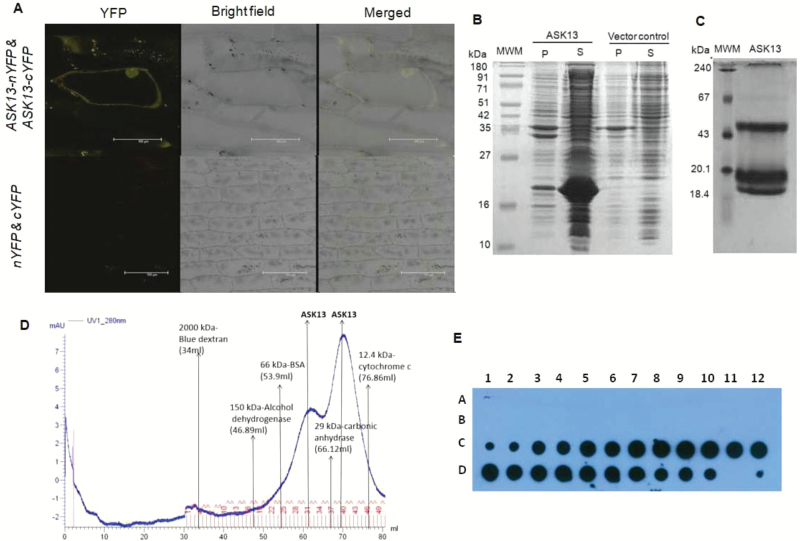
Interactions and biochemical analysis of ASK13. (A) Self-interaction analysis of ASK13 using bimolecular fluorescence complementation assays (BiFC) in onion epidermal cells. The bottom panel shows the negative control, where empty BiFC vectors (cYFP + nYFP) were co-transfected. (B) 12% SDS PAGE analysis of the ASK13 protein over-expressed in *E. coli* BL21 (DE3). Vector control indicates induced cells transformed with the pET23d empty vector; MWM, molecular weight marker; P, pellet fraction; S, soluble fraction. (C) Native PAGE analysis of ASK13. The 10% native polyacrylamide gel shows protein bands of purified ASK13 corresponding to 43 kDa and 19 kDa. The gel was stained with Coomassie Brilliant Blue, destained, and photographed. (D) Size-exclusion chromatography on a Superose 6 column showing the homo-oligomeric nature of purified ASK13. The purified protein and appropriate molecular weight markers were run and the ratio of the elution time to void volume (*V* _e_/*V* _o_) was plotted against the molecular mass on a log scale and fitted using the exponential function *y*=512304*e* ^−4.306*x*^. The approximate molecular mass of ASK13 was calculated using this formula, and was found to be 47.5 kDa and 21.2 kDa. The numbered fractions highlighted in red (31–80 ml) were analysed further for dot blot analysis (after sample loading, the first 30 ml passed through the column). (E) Dot blot analysis with an anti-His antibody of the protein fractions 31–78 eluted using size-exclusion chromatography. A1 in the blot corresponds to fraction 31 and D12 corresponds fraction 78.

Based on the subunit composition and stoichiometry of SCF–E3 complexes, SKP1-like is a monomeric protein. However, Y2H library screening and subsequent BiFC analysis showed that ASK13 was able to interact with itself (Fig. 3A, Table 1). This observation prompted us to examine the nature of the ASK13 protein. Recombinant ASK13 was expressed as a His-tag fusion protein in *E. coli* BL21 DE3 (Fig. 3B). The protein that was expressed and remained in the soluble fractions was purified to near-homogeneity using Ni-affinity chromatography. To determine the subunit composition, size-exclusion chromatography was performed using the purified recombinant his-tagged ASK13 protein. The protein was eluted as two peaks corresponding to molecular masses of 47.5 and 21.2 kDa, which are approximately the dimeric and monomeric masses of the recombinant ASK13 protein (Fig. 3D). The presence of proteins in these fractions was also confirmed by dot blot analysis using an anti-his antibody (Fig. 3E). These analyses suggested that ASK13 exists in at least a monomeric and dimeric form. To further confirm the monomeric and dimeric nature of ASK13, purified recombinant protein was run on a native PAGE gel and two distinct bands corresponding to approximately 18 kDa and 51 kDa were observed (Fig. 3C). Each protein band was excised from the gel and identified and confirmed by MALDI-TOF analysis (Supplementary Fig. S3). These analyses again confirmed the monomeric and homo-dimeric association of the ASK13 protein.

To check the *in vivo* existence of such monomeric and oligomeric associations of ASK13 in Arabidopsis, we performed a size-exclusion chromatography analysis using total proteins extracted from transgenic lines expressing the GFP-fused ASK13 protein. The fused protein was detected in eluted fractions through dot blot analysis using an anti-GFP antibody. The results showed that GFP-fused ASK13 proteins were eluted corresponding to monomeric to dimeric sizes (Supplementary Fig. S4). This does not necessarily confirm the homo-oligomeric nature of the ASK13 protein, as it also interacts with other proteins; however, the determination of self-interaction of the ASK13 protein through Y2H and BiFC analyses, together with the results of the size-exclusion chromatography and the native PAGE analysis of the purified recombinant ASK13 protein suggests that it not only exists in a monomeric form but also in a homo-oligomeric form.

Given that we had observed the self-interaction of ASK13 proteins through Y2H and BiFC (Fig. 3A), and as a recombinant protein *in vitro* (Fig. 3D, 3E) and as a chimeric protein *in vivo* (Supplementary Fig. S4), we were interested to examine whether it could interact with other ASK proteins. Several *ASK* cDNAs were therefore amplified, cloned in both the pDEST-GBKT7 and pDEST-GADT7 vectors, and Y2H analyses were performed. The results showed that some of the members of the ASK gene family exhibited self-interaction and also interacted with other ASK proteins (Table 2, Supplementary Fig. S5).

**Table 2.** Interactions among ASK members as determined by yeast two-hybrid assays

	ASK 1	ASK 2	ASK 3	ASK 4	ASK 5	ASK 6	ASK 7	ASK 8	ASK 9	ASK 10	ASK 11	ASK 13	ASK 14	ASK 19
**ASK 1**												**+**		
**ASK 2**							**+**							
**ASK 3**														
**ASK 4**														
**ASK 5**														
**ASK 6**						**+**	**+**							
**ASK 7**		**+**				**+**	**+**							
**ASK 8**														
**ASK 9**														
**ASK 10**										**+**				
**ASK 11**														
**ASK13**	**+**											**+**		
**ASK 14**														
**ASK 19**														

### Overexpression of *ASK13* enhances seed germination and seedling growth under abiotic stress

**Fig. 4.**
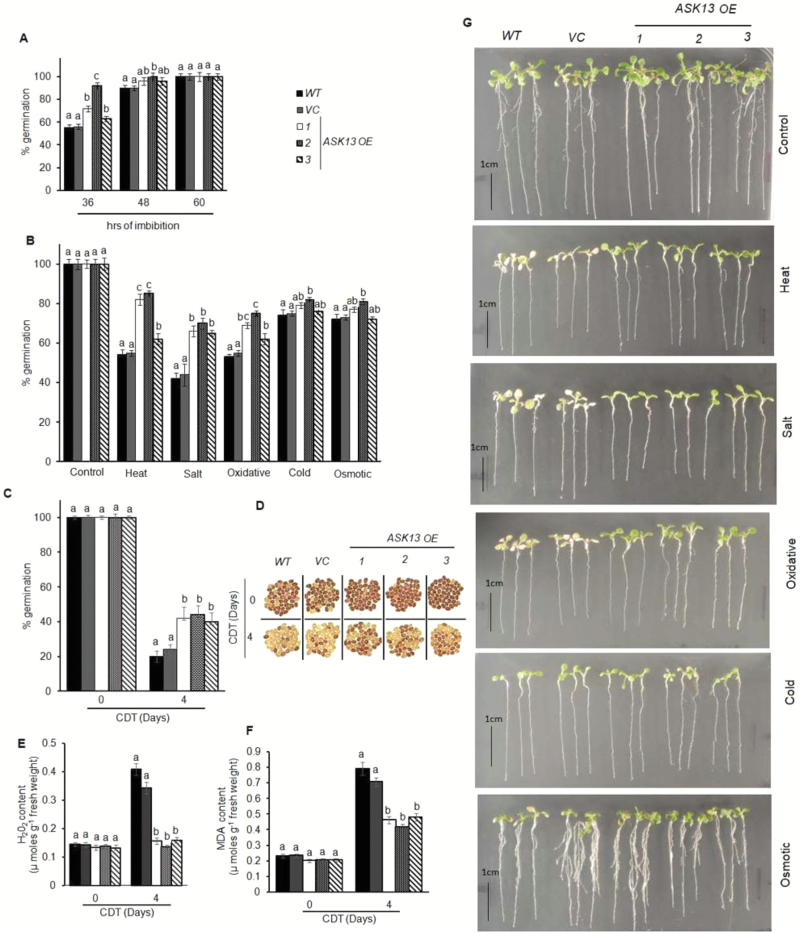
*ASK13*-overexpression results improved Arabidopsis seed germination, vigor, and seedling growth under abiotic stress conditions. Results from three representative independent transformed lines are shown (*ASK13*-OE 1–3). Seeds that had been after-ripened for 8 weeks were used for these experiments, and all data are means (±SD) of four replicates with 50 seeds each. (A) Germination percentage of wild-type (*WT*), empty vector (*VC*), and *ASK13*-transformed seeds. (B) Comparison of germination percentage among seeds under various stress conditions. (C) Germination percentage of seeds without (0 d) or with controlled deterioration treatment (CDT) applied for 4 d. Germination was scored after 7 d of imbibition following the CDT. (D) Viability of seeds with or without CDT, analysed using tetrazolium staining: dark red indicates viable seeds. The images of each genotype were taken separately. Quantitative analysis of (E) H_2_O_2_ and (F) malondialdehyde (MDA) content in the seeds with or without CDT. In the graphs, different letters indicate significant differences among means according to Duncan’s Multiple Range Test (*P*<0.01). (G) Phenotypes of seedlings under various forms of abiotic stress. Stress treatments were applied to 7-d-old seedlings: cold stress, 4 °C; osmotic stress, PEG, –0.5MPa; heat stress, 37 °C; salinity stress, NaCl, 150 mM; and oxidative stress, 2 μM paraquat.

We had observed that siliques and seeds accumulated the highest levels of *ASK13* transcripts and that *ASK13* was up-regulated during seed germination. This prompted us to study the involvement of *ASK13* in seed germination. We therefore generated several independent transgenic Arabidopsis lines constitutively overexpressing *ASK13* through the CaMV 35S promoter. *ASK13*-transformed plants were initially screened for kanamycin resistance and expression of the GUS reporter gene. The presence of the *ASK13* gene in the screened transgenic lines was then confirmed by PCR analysis using *ASK13* and 35S promoter-specific primers Over-accumulation of *ASK13* transcript was then checked in the selected lines (Supplementary Fig. S6), and the lines exhibiting a 3 kan^R^:1 kan^S^ segregation pattern in their progeny were selected. Finally, three independent homozygous T3 lines for *ASK13*-overexpression (OE) were selected and used to assess their germination efficiency. After 36 h of imbibition *ASK13*-OE seeds had completed germination significantly earlier than the Col-0 wild-type and vector control seeds (Fig. 4A, Supplementary Fig. S12).

The germination efficiency of *ASK13* transgenic and control seeds were also compared under various abiotic stress conditions. After 7 d of imbibition, the final germination percentages of the wild-type, vector control, and *ASK13*-transformed lines were similar when tested on normal MS media. However, germination of wild-type seeds was found to be significantly inhibited at high temperature and on media supplemented with NaCl, paraquat, or PEG, whereas germination of the transformed lines was significantly less affected under similar conditions (Fig. 4B). The overexpression lines generally completed germination significantly more than the wild-type or vector control seeds.

We also evaluated the seed germination vigor of the *ASK13*-transformed seeds using a control deterioration treatment (CDT), which has been used effectively for rapid assessment and prediction of seed vigor and longevity in previous studies (Delouche and Baskin, 1973; TeKrony and Hunter, 1995; Ogé *et al.*, 2008). Seeds were subjected to CDT for 0–4 d and germination percentage was then scored. As expected, the *ASK13*-transformed, wild-type, and vector control lines showed no differences in germination before the CDT; however, after 4 d of treatment control seeds exhibited poor germination (20–24%), which was in contrast to the *ASK13*-transformed seeds, where remarkably 40–45% germination was observed (Fig. 4C). A tetrazolium (TZ) assay was performed to assess the seed viability before and after CDT (Berridge *et al.*, 1996). As expected under control conditions, seeds from all genotypes were uniformly stained dark red; however, after CDT only a significant number of the *ASK13*-transformed seeds showed dark-red staining. This was in contrast to control seeds, which remained unstained or were stained pale red (Fig. 4D). In addition, *ASK13* transcript abundance in wild-type seeds was found to be up-regulated during CDT (Supplementary Fig. S7). Previous studies have shown that reduction of germination efficiency and viability during ageing is associated with excess accumulation of ROS in seeds (Bailly, 2004). Therefore, to examine whether the improved seed vigor of the *ASK13*-OE lines was associated with reduced accumulation of ROS and subsequent reduced lipid peroxidation, we analysed H_2_O_2_ and malondialdehyde (MDA) levels in seeds before and after CDT, as they are reliable indicators of ROS stress (Smirnoff, 1995). The results showed that the *ASK13*-OE lines exhibited significantly reduced H_2_O_2_ and MDA accumulation as compared to control lines after CDT (Fig. 4E, F).

To investigate whether *ASK13* plays a role in seedling growth, particularly under environmental stress conditions, growth patterns among the genotypes were compared in 7-d-old seedlings under various abiotic stress conditions. Under control conditions, the*ASK13*-OE, vector control, and wild-type seedlings could not be distinguished (Fig. 4G). In contrast, growth of the vector control and *ASK13*-OE lines differed significantly under stress, particularly under heat, salt, and paraquat-induced oxidative stress conditions. The *ASK13*-OE lines exhibited improved resistance against salinity, heat, and oxidative stress compared with that of the wild-type and vector control lines, which were severely affected in these stress conditions. The growth performance of the *ASK13*-OE seedlings was comparatively superior and they remained relatively green and healthy. Furthermore, the *ASK13*-OE lines exhibited significantly reduced H_2_O_2_ and MDA accumulation as compared to control lines after stress treatment (Supplementary Fig. S8).

### An *ask13* T-DNA insertion mutant and RNAi lines exhibit reduced germination and hypersensitivity to abiotic stress

**Fig. 5.**
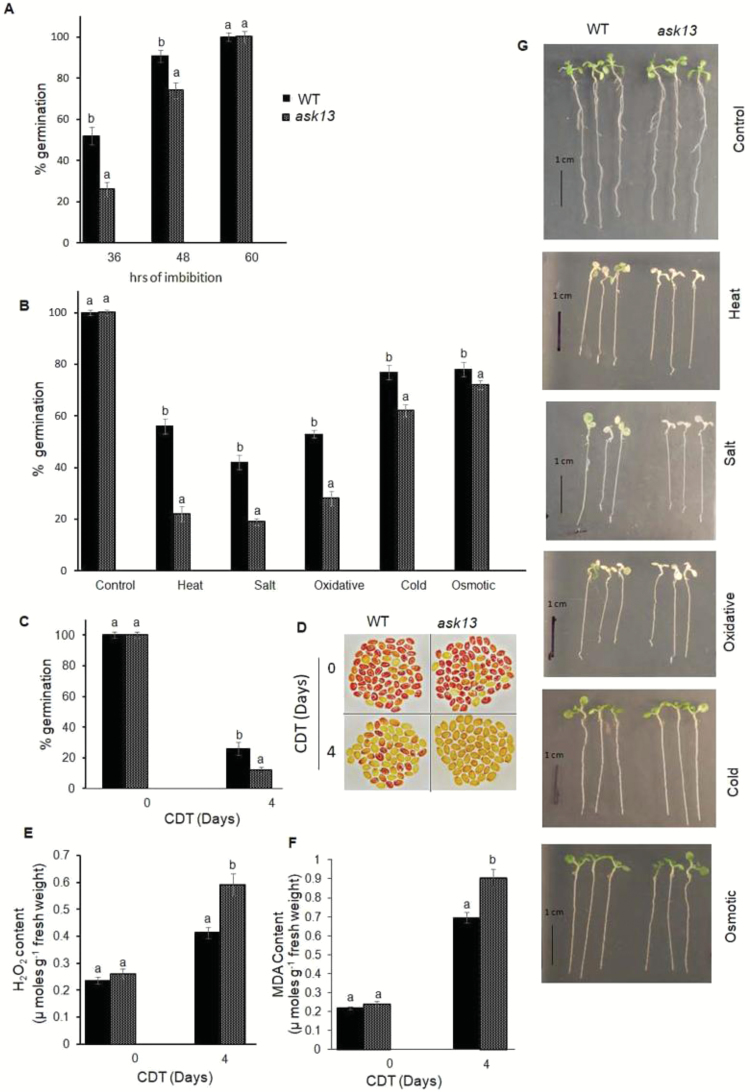
The *ask13* knockdown mutant of Arabidopsis shows reduced seed germination, vigor, and seedling growth under abiotic stress conditions. Seeds that had been after-ripened for 8 weeks were used for these experiments, and all data are means (±SD) of four replicates with 50 seeds each. (A) Germination percentage of wild-type (WT) and *ask13* knockdown seeds. (B) Germination percentage under various stress conditions. (C) Germination percentage without (0 d) or with controlled deterioration treatment (CDT) applied for 4 d. Germination was scored after 7 d of imbibition following the CDT. (D) Viability of seeds with or without CDT, analysed using tetrazolium staining: dark red indicates seeds are viable. The images of each genotype were taken separately. Quantitative analysis of (E) H_2_O_2_ and (F) malondialdehyde (MDA) content in seeds with or without CDT. In the graphs, different letters indicate significant differences among means according to Duncan’s Multiple Range Test (*P*<0.01). (G) Phenotypes of seedlings under various forms of abiotic stress. Stress treatments were applied to 7-day-old seedlings: cold stress, 4 °C; osmotic stress, PEG, –0.5MPa; heat stress, 37 °C; salinity stress, NaCl, 150mM; and oxidative stress, 2 μM paraquat.

Given that *ASK13*-OE seeds exhibited improved seed germination and vigor, we assessed germination when *ASK13* expression was repressed by examining *ask13* (CS466214 –T DNA insertion lines) mutant lines obtained from the GABI-Kat seed collection. The T DNA is inserted in the 3′-UTR region of the *ASK13* gene in this mutant line, which accumulates significantly less *ASK13* transcripts than wild-type plants (Supplementary Fig. S9). Germination analyses showed that this mutant exhibited delayed germination as compared to the wild-type (Fig. 5A, Supplementary Fig. S12). After 36 h of imbibition, 50% of the wild-type seeds were able to complete germination, whereas *ask13* mutant seeds exhibited only 20–30% germination. Differences in germination were still apparent even after 48 h of imbibition; however, after 60 h the seeds from both genotypes completed 100% germination. To further confirm that the slower germination of the *ask13* mutant was due to the reduced expression of *ASK13*, we performed a genetic complementation study where *ASK13* was ectopically expressed in *ask13* mutant plants. Mutants transformed with *ASK13* exhibited restored and improved seed germination, while only the vector without *ASK13* failed to restore the delayed germination phenotype (Supplementary Fig. S10). The role of *ASK13* in seed germination was thus clearly established.

We examined the germination efficiency of *ask13* mutant seeds subjected to various abiotic stress conditions and found that they exhibited significantly reduced germination compared to wild-type seeds under heat, salt, and oxidative stress conditions (Fig. 5B). *ask13* mutant seeds also showed reduced germination under cold and osmotic stress.

As the *ASK13*-OE seeds were found to exhibit improved seed vigor, we also examined this in the mutant seeds. After 4 d of CDT, 20–30% of the wild-type seeds completed germination, whereas only10–15% of the *ask13* mutant seeds were able to do so (Fig. 5C). The TZ assay also demonstrated the reduced viability of the *ask13* seeds after CDT (Fig. 5D). These analyses confirmed that the *ask13* mutant seeds were hypersensitive to ageing. We also analysed ROS and MDA levels and found that the *ask13* mutant seeds accumulated significantly more ROS and MDA than the wild-type seeds after CDT (Fig. 5E, F). Growth responses were evaluated in 7-d-old seedlings that were subjected to various forms of abiotic stress. Under control conditions, the wild-type and *ask13* mutant seedlings grew normally and did not show any noticeable differences (Fig. 5G). However, under heat, salt, and oxidative stress conditions, growth of the *ask13* mutant seedlings was severely affected, although the extent of the growth inhibition differed between treatments. In contrast, wild-type seedlings were comparatively less affected and their growth performance was better. In addition, the *ask13* transgenic lines exhibited significantly increased H_2_O_2_ and MDA accumulation compared with the wild-type after the stress treatments (Supplementary Fig. S8).

**Fig. 6.**
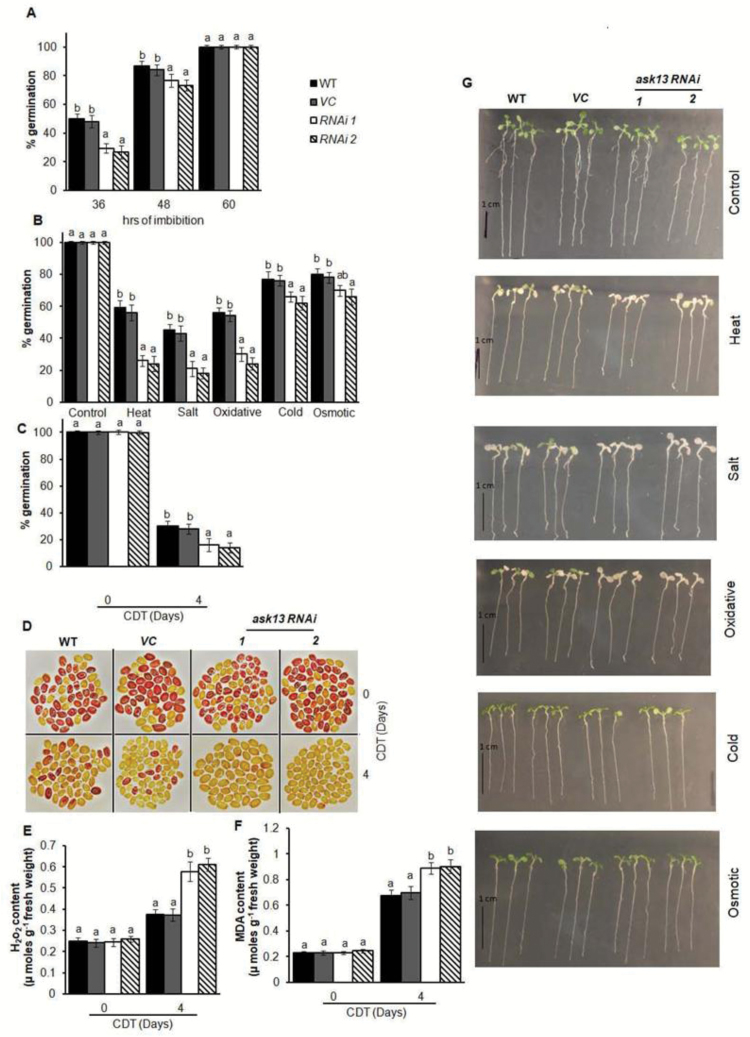
The *ask13*-RNAi lines show reduced seed germination, vigor, and seedling growth under abiotic stress conditions. Data from two representative independent *ask13*-RNAi transformed lines are shown. (A) Germination percentage for the wild-type (WT), vector control (*VC*), and *ask13*-RNAi seeds. (B) Germination percentage under various stress conditions. (C) Germination percentage without (0 d) or with controlled deterioration treatment (CDT) applied for 4 d. Germination was scored after 7 d of imbibition following the CDT. In (A–C) data are means (±SD) of four biological replicates with 50 seeds each. (D) Viability of seeds with or without CDT, analysed using tetrazolium staining: dark red indicates seeds are viable. The images of each genotype were taken separately. Quantitative analysis of (E) H_2_O_2_ and (F) malondialdehyde (MDA) content in seeds with or without CDT; data are means (±SD) of four biological replicates. In the graphs, different letters indicate significant differences among means according to Duncan’s Multiple Range Test (*P*<0.01). (G) Phenotypes of seedlings under various forms of abiotic stress. Stress treatments were applied to 7-d-old seedlings: cold stress, 4 °C; osmotic stress, PEG, –0.5MPa; heat stress, 37 °C; salinity stress, NaCl, 150mM; and oxidative stress, 2 μM paraquat.

To further validate the data from the *ask13* mutants, independent RNAi lines of *ask13* were generated. Knockdown expression of these lines was confirmed by transcript analysis (Supplementary Fig. S11). In common with the *ask13* mutant, *ask13*-RNAi seeds also exhibited delayed germination when compared to the wild-type and vector control seeds (Fig. 6A, Supplementary Fig. S12). Only 20–30% of the *ask13*-RNAi seeds had germinated after 36 h of imbibition, compared with 40–50% for the wild-type and vector control seeds. The germination efficiency of the *ask13*-RNAi seeds was examined under various abiotic stress conditions, and was found to be significantly reduced in all cases compared to the wild type and vector control seeds (Fig. 6B). In common with the *ask13* mutant seeds, the germination vigor of the *ask13*-RNAi seeds was also compromised. After 4 d of CDT, 20–30% of the wild-type and vector control seeds had germinated, whereas only 10–20% of the *ask13*-RNAi seeds had done so (Fig. 6C). The TZ assay also demonstrated the reduced viability of the *ask13*-RNAi seeds after CDT (Fig. 6D). In common with the *ask13* mutant, the *ask13*-RNAi seeds also accumulated significantly more ROS and MDA than the wild-type and vector control seeds after CDT (Fig. 6E, F) and the growth performance of the seedlings was also more compromised under various forms of abiotic stress (Fig. 6G, Supplementary Fig. S8). Overall, the functional analyses of *ask13* T-DNA insertion mutant and the RNAi lines confirmed that ASK13 positively influences seed germination and seedling growth, particularly under abiotic stress conditions.

## Discussion

The SKP1 protein, one of the four members of any SCF–E3 ligase complex, acts as an adapter that binds cullin on one side and various F-box proteins on the other side and thus mediates the addition of ubiquitin to diverse cellular proteins, and it is thereby involved in various biological processes (Bai *et al.*, 1996). In the present study, we have shown that ASK13 not only binds to F-box proteins but also interacts with several other proteins such as ATPase F0/V0 complex subunit C protein, DNA topoisomerase 1 alpha subunit protein, and hsp70 binding protein1, none of which are a component of SCF complexes (Table 1). This suggests that ASK13 is not only a component of SCF–E3 ligase complexes it also but that it also forms various complexes with several other proteins and is likely to participate in different cellular processes in Arabidopsis than simply ubiquitination. Furthermore, these non-SCF proteins were also found to interact with a few other ASK proteins and were not only limited to ASK13 (Fig. 2). This finding suggests that SKP1-like proteins also participate in other cellular processes apart from their participation in ubiquitination in Arabidopsis. Although such interactions of ASK13 with non-SCF complex proteins have not been reported in plants before, similar interactions have been reported in yeast and animal systems. For example, *SKP1* of *S. cereviseiae* forms a complex with RAVE, which is a regulator of (H^+^)-ATPase (V-ATPase) assembly (Seol *et al.*, 2001). In addition, the yeast SKP1 protein also forms a complex called centromere-binding factor 3 (Stemmann *et al.*, 2002) and with the suppressor of G2 allele of SKP1 (SGT1) protein (Willhoft *et al.*, 2017). Our biochemical analysis determined that ASK13 not only exists as a monomeric protein, which is essential for formation of a functional SCF complex, but is also found to exist as homo-oligomeric and heteromeric protein complexes with other ASK proteins (Fig. 3). However, the biological significance of the homo-oligomeric form of ASK13 or its heteromeric association with other ASK proteins in Arabidopsis is unclear at present. It may be speculated that, as only a monomeric form is required to participate in SCF complexes, oligomeric forms of ASK13 may hinder the formation of active complexes, which may eventually affect the overall ubiquitination process of target proteins. However, such a multifaceted regulation of ubiquitination processes through oligomerization of ASK proteins remains to be validated by experimental evidence. Interestingly, some of the F-box proteins, such as FBXW7, FBX4 from humans, and CDC4 from yeast, have been reported to form homo-dimers but their function in *in vivo* remains unclear (Tang *et al.*, 2007; Welcker and Clurman, 2007; Li and Hao, 2010).

Previous reports and our present study have clearly demonstrated that *ASK13* is highly expressed in siliques and seeds and it is also up-regulated during seed germination and in seedlings exposed to abiotic stress (Fig. 1, Takahashi *et al.*, 2004). Seed germination and early seedling growth is largely influenced by environmental and internal cues (Bewley and Black, 1994; Miransari and Smith, 2014; Lu *et al.*, 2016), and the ubiquitin–26S proteasome pathway plays a pivotal role in responding to various external and internal stimuli to facilitate seed germination and post-germination process in various plant species (Kurepa *et al.*, 2009*a*;Song *et al.*, 2012; Karmous *et al.*, 2014). Previous studies have also shown that various members of SCF–E3 ligase complexes play important roles in regulating seed germination and seedling growth by modulating hormone and light signaling pathways (Gagne *et al.*, 2004; Kurepa *et al.*, 2009*b*). Two F-box genes, *SLY* and *SNY*, have been shown to positively influence seed germination by targeting GA-repressor DELLA proteins (McGinnis *et al.*, 2003; Dill *et al.*, 2004; Strader *et al.*, 2004). PIL5/PIF1, a phytochrome-interacting basic helix-loop-helix protein and a key negative regulator of seed germination in Arabidopsis, is degraded through the 26S proteome pathway. Red and far-red light signals trigger PIL5/PIF1 protein degradation to release the inhibition of synthesis of bioactive GA (Oh *et al.*, 2004, 2006; Shen *et al.*, 2005). Several F-box proteins that play a key role in various abiotic stress tolerances have also been characterized (Zhang *et al.*, 2008; Maldonado-Calderón *et al.*, 2012; Zhou *et al.*, 2015). Previous studies have shown that ASK1 plays an important role in flower development and male meiosis in Arabidopsis (Yang *et al.*, 1999, 2006). The possibility of a multifaceted role of ASK1 in plant development has also been suggested, with studies that have demonstrated various potential substrates of ASK1–E3 ligases, which are involved in transcription and post-translational processes (Lu *et al.*, 2016). An ASK13-interacting F-box protein has been reported, which positively regulates seed germination (Majee *et al.*, 2017). Our analyses of *ASK13*-overexpression and knockdown lines confirmed the role of ASK13 in seed germination, seed vigor, and seedling growth, particularly under abiotic stress (Figs 4, 5). *ASK13*-OE lines were able to mitigate stress-induced inhibition of seed germination and seedling growth, while the *ask13* knockdown mutant exhibited delayed germination and hypersensitivity to stress. It is likely that through interactions with different F-box proteins, ASK13 can potentially form numerous functional SCF–E3 complexes that potentially modulate the abundance and activity of various regulatory proteins through ubiquitination and play a key role in facilitating seed germination or seedling growth, particularly under abiotic stress conditions. Interestingly, several F-box proteins (At1g08710, At1g23780, At5g49000, At5g45360, At3g52030) that interact with ASK13 are also predominantly expressed in seeds Supplementary Fig. S2. The similar expression patterns between F-box proteins and ASK13 further adds to the possibility of several potential ASK13–E3s that may be implicated in regulating seed germination and seedling growth under abiotic stress conditions, possibly through modulating ROS accumulation.

In addition to this possibility, *ASK13* may also positively influence seed germination and seedling growth by modulating the non-SCF complex proteins by unknown mechanisms, since several interacting non-SCF proteins, such as ATPase F0/V0 complex subunit C protein and HSP70 binding protein 1, have been implicated in germination and seedling growth under abiotic stress (Cooley *et al.*, 1999; Zhang *et al.*, 2010). Furthermore, several non-F-box proteins were also found to express along with *ASK13* in seed and seedlings Supplementary Fig. S2.

In conclusion, our data strongly suggest that ASK13 is not only a member of SCF complexes, but is also implicated in different cellular processes and positively regulates seed germination and seedling growth under abiotic stress conditions. In addition, our results raise the intriguing possibility of the involvement of SKP1-like proteins in multifaceted regulation in plant development.

## Supplementary data

Supplementary data are available at *JXB* online.

Fig. S1. *ASK13* promoter-GUS analysis and sub-cellular localization.

Fig. S2. Expression profiles of genes encoding proteins that interact with ASK13.

Fig. S3. Mascot Search results for ASK13 isolated from native PAGE protein bands.

Fig. S4. Dot blot analysis of eluted fractions from size-exclusion chromatography.

Fig. S5. Yeast two-hybrid analysis of ASK proteins.

Fig. S6. qRT-PCR analysis of *ASK13* transgenic lines.

Fig. S7. qRT-PCR analysis of *ASK13* in seeds after controlled deterioration treatment.

Fig. S8. Stress responses of *ASK13*-overexpression, *ask13* T-DNA insertion mutants, and *ask13*-RNAi seedlings.

Fig. S9. Characterization of the *ask13* mutant.

Fig. S10. *ask13* mutant complementation analysis.

Fig. S11. qRT-PCR analysis of *ASK13* in the *ask13*-*RNAi* transgenic lines.

Fig. S12. Germination analysis of *ASK13*-overexpression, *ask13* mutant, and *ask13*-RNAi lines.

Table S1. List of primers.

Supplementary Figures and Tables
