# Supplementary material for: Arabidopsis SKP1-like protein13 (ASK13) positively regulates seed germination and seedling growth under abiotic stress
Source: J Exp Bot. 2018 May 18;69(16):3899–915. doi: 10.1093/jxb/ery191 (PMC6054272; doi:10.1093/jxb/ery191)
Supplement: Supplementary Figures and Tables [file ery191_suppl_supplementary_figures_tables.pdf]

Supplementary data: Rao et al.,

Arabidopsis SKP1-like protein 13 (ASK13) positively regulates seed germination and seedling growth under abiotic stresses.

A

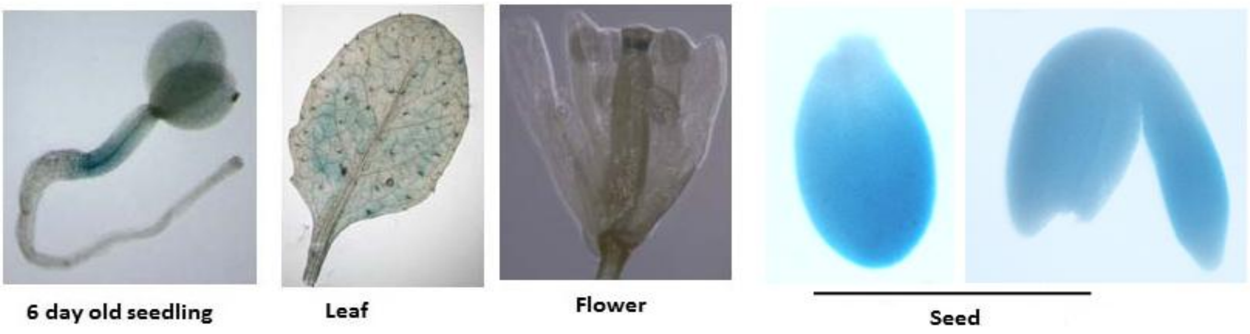

B

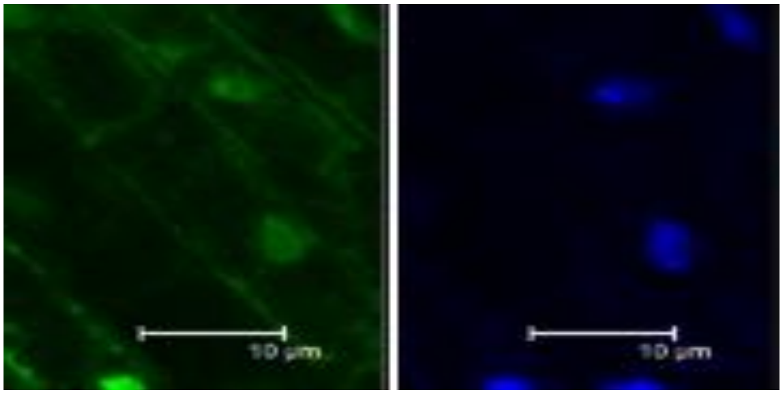

Supplementary Figure S1. A) Histochemical localization of GUS activity in seedling (six-day old), leaf from six weeks old mature plant, mature opened flower and after ripened seed of transformed Arabidopsis *ASK13* promoter-GUS fusion lines. B) Sub-cellular localization of ASK13. Localization of ASK13: GFP in roots of 5 day old Arabidopsis transgenic seedlings. Nuclei were stained with DAP

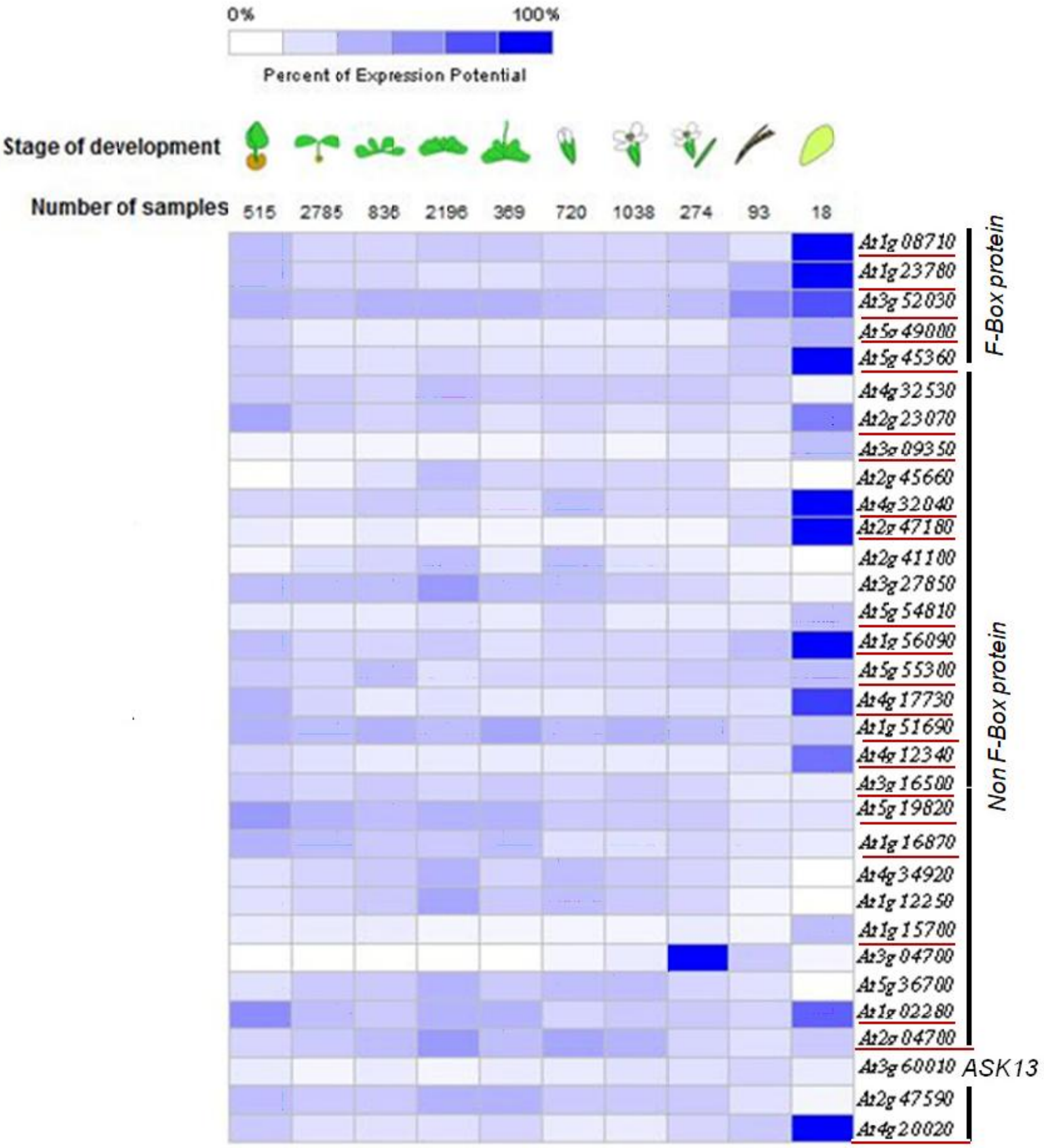

Supplementary Figure S2. Expression profiles of 32 ASK13 interacting F-box, Non F-box protein coding genes were analyzed using affymetrix expression microarrays of genevestigator database (Hruz et al., 2008). Red underlined locus identification codes denotes higher expression in seed.

A

Mascot Search Results

MATRIX SCIENCE

User : **uma**
Email : **uma0200@gmail.com**
Search title : **D:\Analyst Data\Projects\Default\Data\2014\15-12-14\ Ask13 mono.wiff (sample number 1)**
MS data file : **D:\Mascot temp files\mas60A.tmp**
Database : **NCBInr 20141208 (53438708 sequences; 19231914498 residues)**
Taxonomy : **Arabidopsis thaliana (thale cress) (65585 sequences)**
Timestamp : **16 Dec 2014 at 05:18:04 GMT**
Protein hits : **gi|18411415** SKP1-like protein 13 [Arabidopsis thaliana]

Mascot Score Histogram

Ions score is -10*Log(P), where P is the probability that the observed match is a random event.
Individual ions scores > 32 indicate identity or extensive homology (p<0.05).
Protein scores are derived from ions scores as a non-probabilistic basis for ranking protein hits.

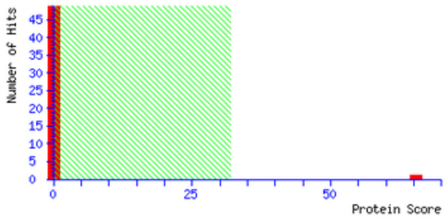

Peptide Summary Report

Format As | Peptide Summary | Help
Significance threshold p< 0.05 | Max. number of hits AUTO
Standard scoring | MudPIT scoring | Ions score or expect cut-off 0 | Show sub-sets 0
Show pop-ups | Suppress pop-ups | Sort unassigned Decreasing Score | Require bold red
Preferred taxonomy All entries

Select All | Select None | Search Selected | **Error tolerant**

1. gi|18411415 **Mass:** 17394 **Score:** 65 **Matches:** 6(2) **Sequences:** 5(2) **emPAI:** 0.46
SKP1-like protein 13 [Arabidopsis thaliana]
Check to include this hit in error tolerant search

Query	Observed	Mr(expt)	Mr(calc)	ppm	Miss	Score	Expect	Rank	Unique	Peptide
105	471.7463	941.4780	941.3953	87.8	0	31	0.16	1	U	K.WDAEFMK.A + Oxidation (M)
113	527.7936	1053.5726	1053.4953	73.4	1	39	0.018	1	U	K.KWDAEFMK.A
115	535.7893	1069.5640	1069.4903	69.0	1	(8)	31	1	U	K.KWDAEFMK.A + Oxidation (M)
287	1052.5223	2103.0300	2103.0007	13.9	0	40	0.02	1	U	R.ALLGIENDFTPEEEEEIR.K
307	744.7480	2231.2222	2231.0957	56.7	1	31	0.13	1	U	R.ALLGIENDFTPEEEEEIRK.E
375	1338.5000	5349.9709	5349.5765	73.7	0	23	0.28	1	U	K.MVMLLSSDGESFQVEEAVAVQSQTIAHMIEDDCVANGVPIANVTGVILSK.V + 3 Oxidation (M)

B

User : uma
Email : uma0200@gmail.com
Search title : D:\Analyst Data\Projects\Default\Data\2014\15-12-14\Ask13 trimer.wiff (sample number 1)
MS data file : D:\Mascot temp files\mas606.tmp
Database : NCBInr 20141208 (53438708 sequences; 19231914498 residues)
Taxonomy : Arabidopsis thaliana (thale cress) (65585 sequences)
Timestamp : 16 Dec 2014 at 04:30:12 GMT
Protein hits : gi|18411415 SKP1-like protein 13 [Arabidopsis thaliana]
gi|18409740 pyruvate kinase [Arabidopsis thaliana]

Mascot Score Histogram

Ions score is -10*Log(P), where P is the probability that the observed match is a random event.
Individual ions scores > 33 indicate identity or extensive homology (p<0.05).
Protein scores are derived from ions scores as a non-probabilistic basis for ranking protein hits.

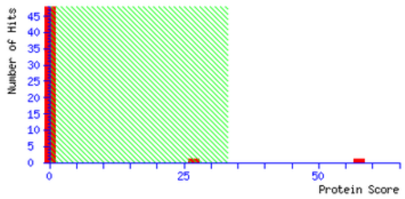

Peptide Summary Report

Format As | Peptide Summary | Help
Significance threshold p< 0.05 | Max. number of hits AUTO
Standard scoring | MudPIT scoring | Ions score or expect cut-off 0 | Show sub-sets 0
Show pop-ups | Suppress pop-ups | Sort unassigned Decreasing Score | Require bold red
Preferred taxonomy All entries

Select All | Select None | Search Selected | Error tolerant

1. gi|18411415 **Mass:** 17394 **Score:** 58 **Matches:** 8(2) **Sequences:** 4(2) **emPAI:** 0.46
SKP1-like protein 13 [Arabidopsis thaliana]
Check to include this hit in error tolerant search

Query	Observed	Mr(expt)	Mr(calc)	ppm	Miss	Score	Expect	Rank	Unique	Peptide
92	463.7523	925.4901	925.4004	96.9	0	(12)	10	2	U	K.WDAEFMK.A
94	471.7483	941.4821	941.3953	92.2	0	25	0.58	1	U	K.WDAEFMK.A + Oxidation (M)
105	527.7871	1053.5596	1053.4953	61.0	1	47	0.0035	1	U	K.KWDAEFMK.A
107	535.7849	1069.5553	1069.4903	60.8	1	(2)	1.2e+02	2	U	K.KWDAEFMK.A + Oxidation (M)
108	535.8002	1069.5859	1069.4903	89.5	1	(8)	25	1	U	K.KWDAEFMK.A + Oxidation (M)
300	744.7403	2231.1990	2231.0957	46.3	1	37	0.034	1	U	R.ALLGIENDFTPEEEEEIRK.E
375	1334.7000	5334.7709	5334.5656	38.5	0	23	0.28	1	U	K.MVMLLSSDGESFQVEEAVAVQSQTIAHMIEDDCVANGVPIANVTGVILSK.V + Deamidated (NQ); 2 Oxidation (M)
376	1338.4227	5349.6618	5349.5765	16.0	0	(12)	3.1	1	U	K.MVMLLSSDGESFQVEEAVAVQSQTIAHMIEDDCVANGVPIANVTGVILSK.V + 3 Oxidation (M)

Supplementary Figure S3: Identification of proteins from native PAGE (A) protein band corresponds to ~ 18kDa (B) protein band corresponds to ~ 51kDa (Fig 3C) by MS analysis.

The protein spots excised from native PAGE gel were initially subjected to in-gel trypsin digestion. Protein identification and peptide analysis was carried out on a 4800 MALDI-TOF/TOF (Applied Biosystems/ MDS SCIEX) at NIPGR proteomics facility as described in Kaur et al. (2015).

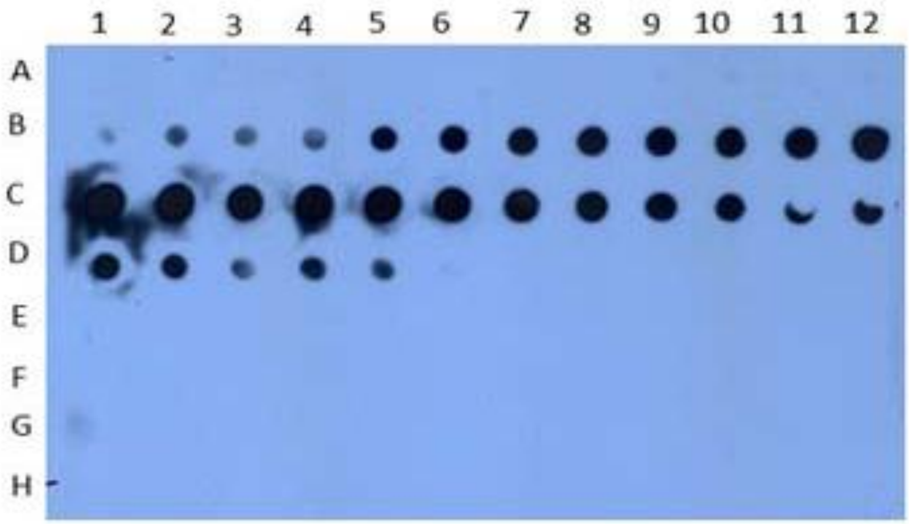

Supplementary Figure S4. Dot blot analysis of protein in eluted fractions from size exclusion chromatography. Approximate molecular weight of GFP fused ASK13 protein in fractions detected through dot blot with anti-GFP antibody. The first 12 fractions /samples contained no ASK protein. Thereafter, fractions contained varying amounts of the chimeric protein until fraction 41 at which point, no GFP Ab-reactive protein was detected in the dot blot.

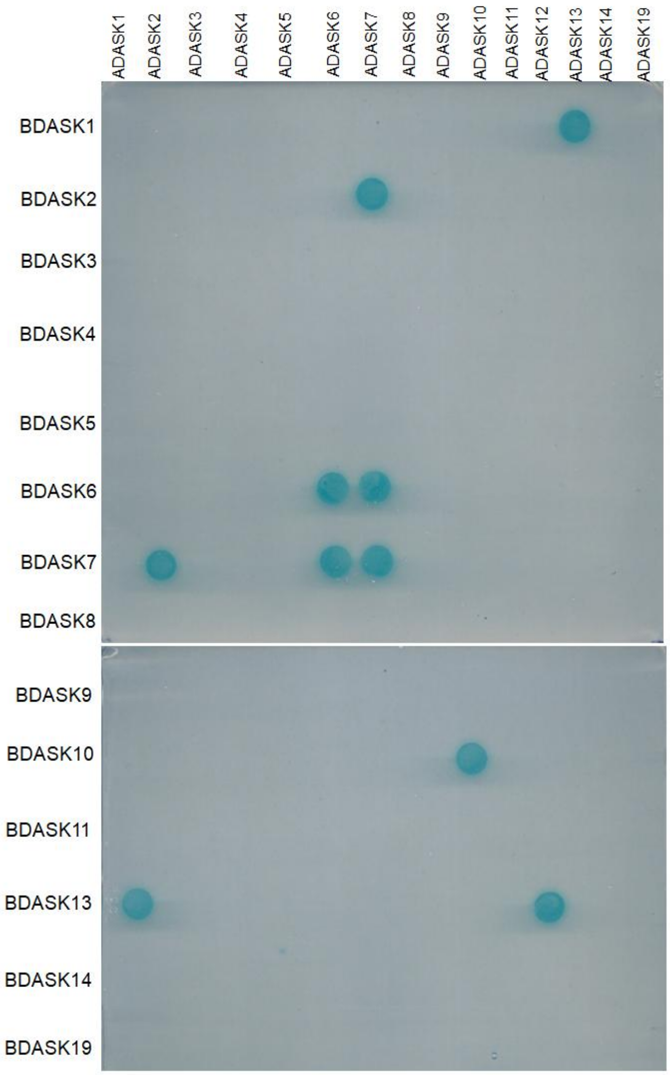

Supplementary Figure S5. Y2H interactions among ASK proteins. Yeast cells co-transformed with indicated pDEST-GBKT7: *ASKs* and pDEST-GADT7: ASK cDNAs were grown on yeast synthetic drop-out medium lacking leucine, tryptophan, histidine, and adenine (QDO) supplemented with X-α-Gal and aureobasidin A.

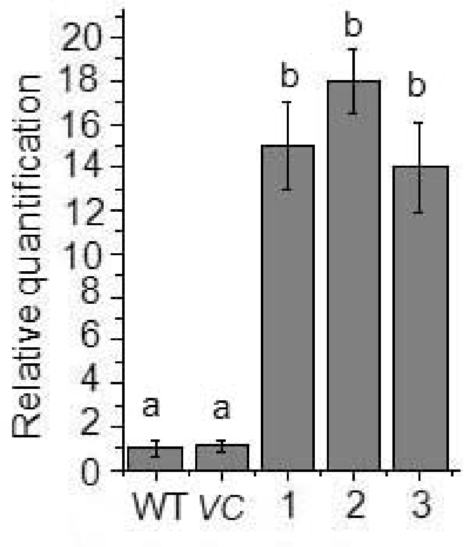

Supplementary Figure S6. Quantitative RT-PCR analysis of *ASK13* in transgenic lines. Total RNA from each sample was reverse transcribed and subjected to real time PCR analysis. The relative expression value of *ASK13* was normalized to an endogenous control *18S* ribosomal small subunit RNA and calculated using the ΔΔCT method (Applied Biosystems). Values are the result of triplicate analysis of three biological replicates. Error bars indicate the standard deviation. Significant differences among means (α=0.01) are denoted by different letters.

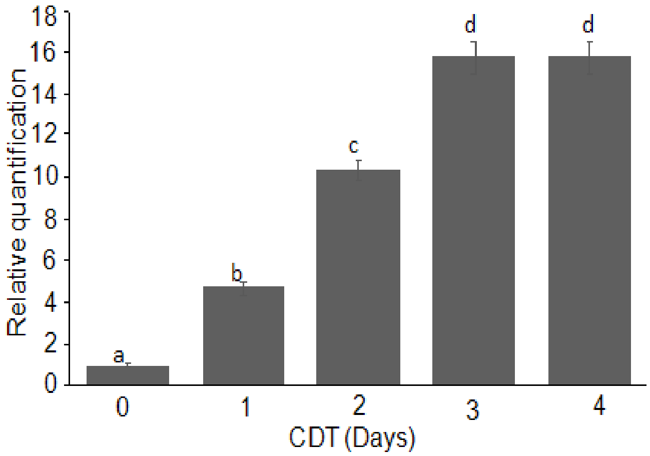

Supplementary Figure S7. Quantitative RT-PCR analysis of *ASK13* transcript after CDT treatment of wild type seeds. The relative expression value of *ASK13* was normalized with an endogenous control *18S* ribosomal small subunit RNA and calculated using the ΔΔCT method (Applied Biosystems). Values are the result of triplicate analysis of three biological replicates. Error bars indicate the standard deviation. Significant differences among means (α=0.01) are denoted by different letters.

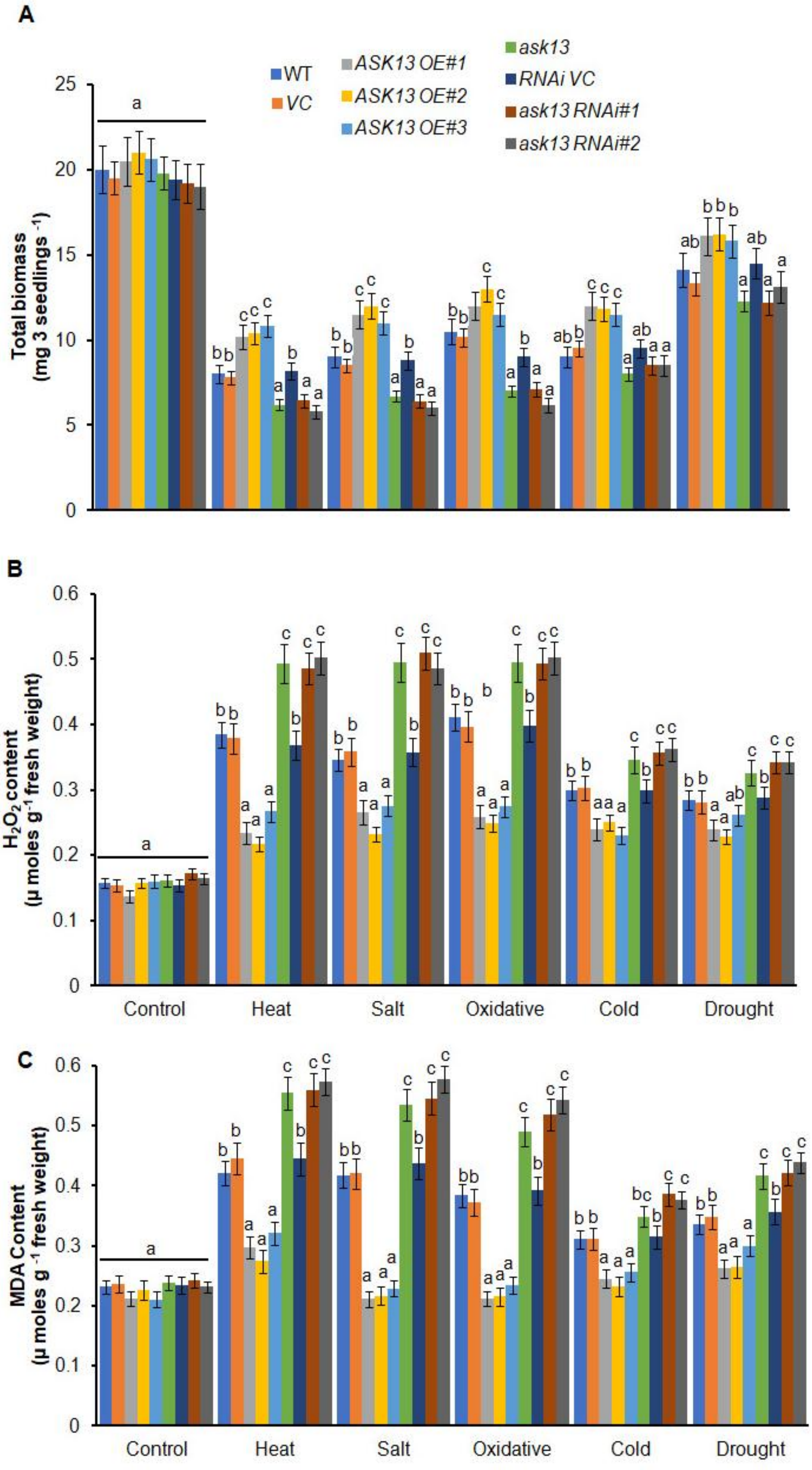
A
WT
VC
ASK13 OE#1
ASK13 OE#2
ASK13 OE#3
ask13
RNAi VC
ask13 RNAi#1
ask13 RNAi#2
Total biomass
(mg 3 seedlings -1)
B
H2O2 content
(µ moles g-1 fresh weight)
Control
Heat
Salt
Oxidative
Cold
Drought
C
MDA Content
(µ moles g-1 fresh weight)
Control
Heat
Salt
Oxidative
Cold
Drought

Supplementary Figure S8. Stress responses of *ASK13* overexpression, *ask13* T-DNA insertion mutant and *ask13 RNAi* seedlings. Quantitative analysis of (A **)** Biomass (B) H_2O_2 content and (C) MDA of wild type *(wt)*, vector control *(vc), ASK13* overexpression, *ask13* mutant and *ask13 RNAi* seedlings. Quantifications were done after various stress treatments in 14 days old seedlings. Values are mean ± SE of three biological replicates. Significant differences among means (α=0.01) are denoted by different letters.

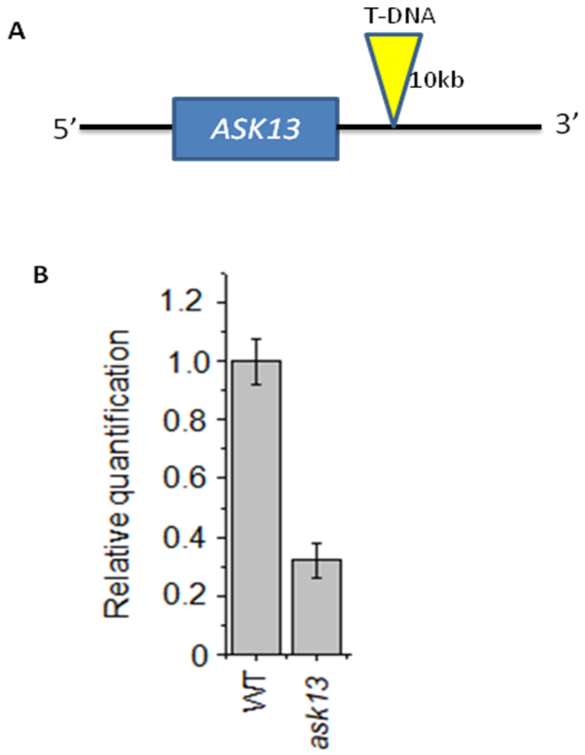

Supplementary Figure S9. A) Schematic representation of T-DNA insertion in *ask13* mutant. B) Quantitative RT PCR analysis of *ASK13* in the *ask13* mutant. Total RNA from the wild type and from mutant was reverse transcribed and subjected to real time PCR analysis. The relative expression value of *ASK13* was normalized to an endogenous control 18S ribosomal small subunit RNA and calculated using the ΔΔCT method (Applied Biosystems). Values are the result of triplicate analysis of three biological replicates. Error bars indicate the standard deviation. Significant differences among means (α=0.01) are denoted by the different letters. (1 to 3 represents three different plants from the same mutant line).

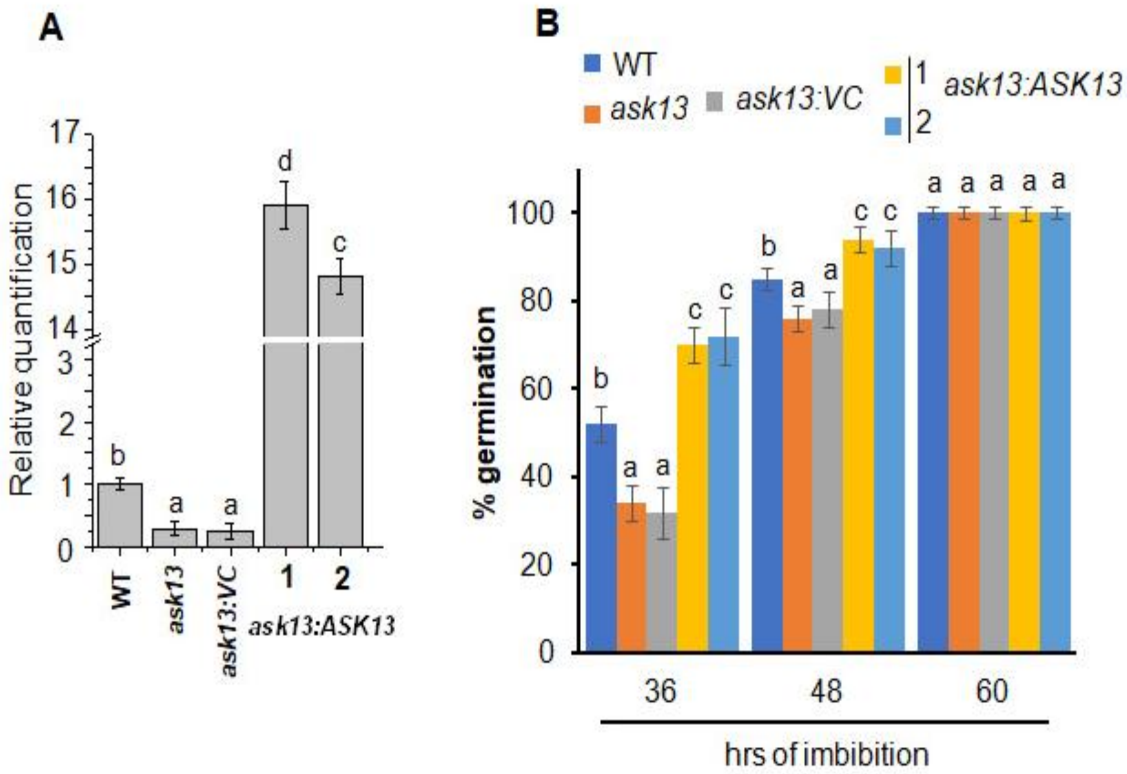

Supplementary Figure S10: Characterization of *ask13* mutant complemented by *ASK13*. *ASK13* was expressed by CaMV 35S promoter in *ask13* mutant line **(A**) *ASK13* transcript accumulation in wild type (WT), *ask13* mutant, *ask13* mutant harboring empty vector *(VC)* and *ask13* mutant harboring *ASK13 (ask13:ASK13)***. (B)** Seed germination analysis of wild type (WT), ask13 mutant, *ask13* mutant harboring empty vector *(VC)*, and *ask13* mutant harboring *ASK13(ask13:ASK13).*

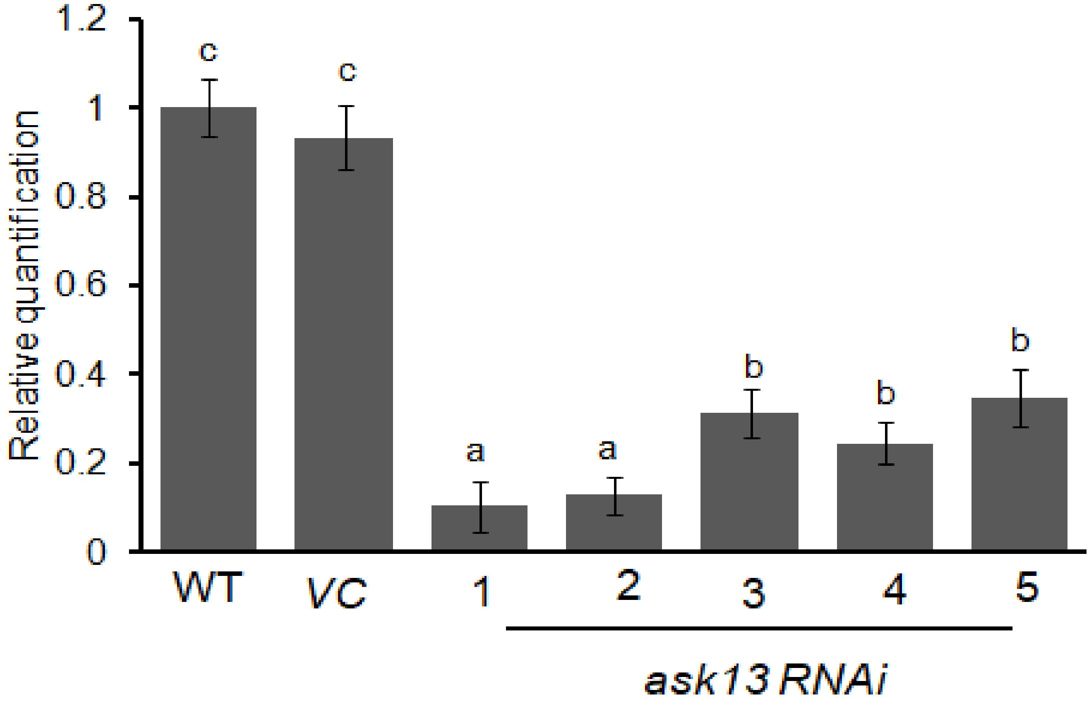

Supplementary Figure S11: Quantitative RT PCR analysis of *ASK13* in the *ask13 RNAi* lines. Total RNA from the wild type, vector control and *ask13 RNAi* lines was reverse transcribed and subjected to real time PCR analysis. The relative expression value of *ASK13* was normalized to an endogenous control 18S ribosomal small subunit RNA and calculated using the ΔΔCT method (Applied Biosystems). Values are the result of triplicate analysis of three biological replicates. Error bars indicate the standard deviation. Significant differences among means (α=0.01) are denoted by the different letters

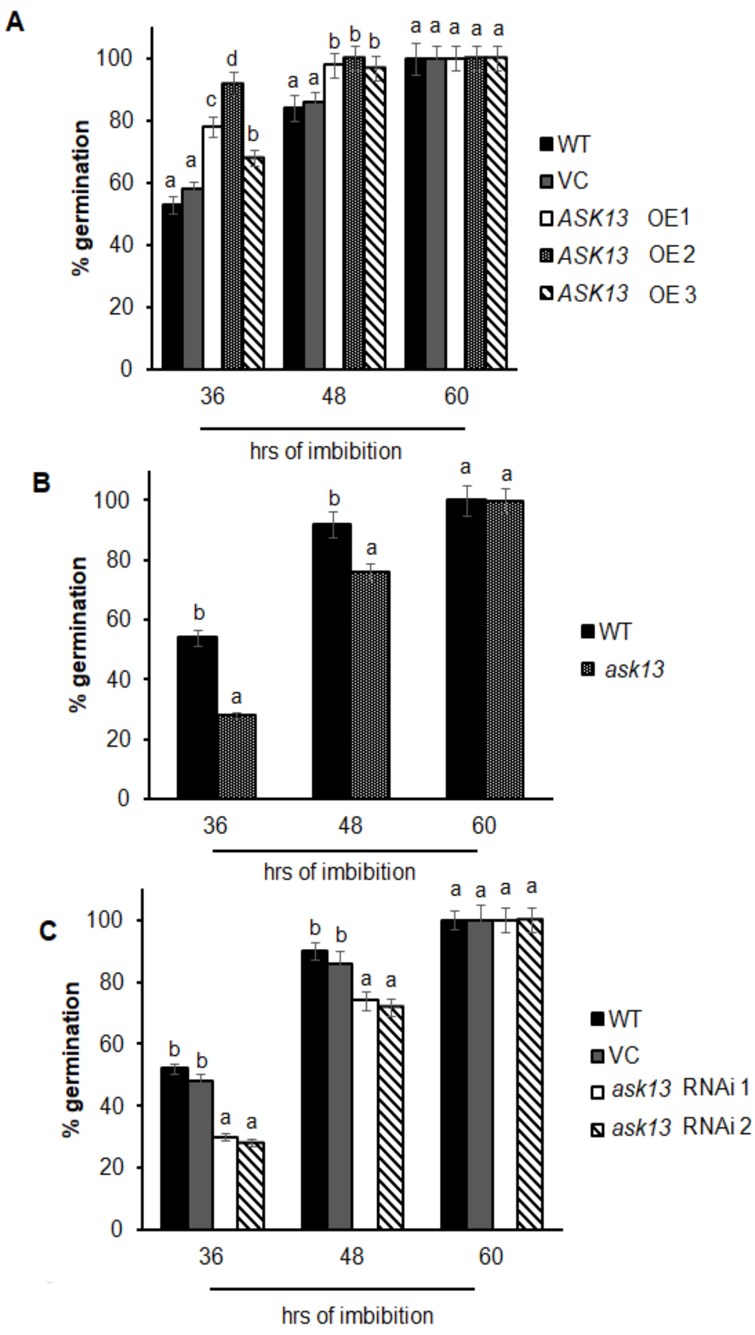

Supplementary Figure S12. Germination percentage of wild type (WT), empty vector (*VC*), and *ASK13* OE, *ask13* mutant *ask13* RNAi lines seeds during the course of germination. Data are means ±SD of four repetitions with 50 seeds each.

Table S1: Primers used in this study

Primer No	Primer sequence 5'- 3'	Purpose
MM 1 F	CGACGTTAGTGTGCTTTCTGATCT	*ASK13* Real time
MM 2 R	ACACATACACACAAGAGAAGAAAGAACA	
MM 3 F	TCCTAGTAAGCGCGAGTCATCA	*18S* rRNA (*At3g41768*) Real time
MM 4 R	CGAACACTTCACCGGATCAT	
MM 5 F	GGAATTCATGTCGAAGATGGTTATGTTGC	*ASK13* pGBKT7BD construct
MM 6 R	CGGATCCTCATTCAAAAGCCCATTGATTC	
MM 7 F	CACCATGTCTGCAAACGAGATACCCGACG	*At1g08710* Gateway entry
MM 8 R	TCAAAGAACAAGCAGATTTTCAGGAATA	
MM 9 F	CACCATGAAGCTACGATTGAGACATCACG	*At1g23780* Gateway entry
MM 10 R	CTAGTTGAGTCCCCCAAGATTGCATC	
MM 11 F	CACCATGGGGAGAACAGAGACCGGCGACG	*At3g52030* Gateway entry
MM 12 R	TTACGAGAGATTGAACTTCCATACACT	
MM 13 F	CACCATGTCGTCTCCAGAGAGGAAGAGGA	*At5g49000* Gateway entry
MM 14 R	TCAAAGAGTAGCAGCAAGAGCACCCAC	
MM 15 F	CACCATGTCTGTTTCCGATGAGGAAGATG	*At5g45360* Gateway entry
MM 16 R	TCAGCAGAACCTCAGGGTACGTTCCAG	
MM 17 F	CACCATGGCGACGATTCCAATGGATATCG	*At4g12560* Gateway entry
MM 18 R	TTATAAGACCAGCTTGAATCCTTTG	
MM 19 F	CACCATGTCTGGTGTGGTTGCTCTTGGTC	*At4g32530 (ATPASE FO/VO COMPLEX SUBUNIT)*
MM 20 R	CTATTTCGTTGGCCATGTCGCTTGT	
MM 21 F	CACCATGGCCTTAAGGCCTTGTACTGGAT	*At2g23070 (PROTEIN KINASE)* Gateway entry
MM 22 R	TCACTGGCTGCGCGGCGTACGGCTG	
MM 23 F	CACCATGGCGAAAGACGGACCTAATTGGG	*At3g09350 (ORTHOLOG OF HUMAN HSBP1)*
MM 24 R	CTATGGTCCAAGTAGTTTCATAGGT	
MM 25 F	CACCATGGTGAGGGGCAAAACTCAGATGA	*At2g45660 (AGAMOUS LIKE 20)* Gateway entry
MM 26 R	TCACTTTCTTGAAGAACAAGGTAAC	
MM 27 F	CACCATGTCGTTTAACAGCTCCCACCTCC	*At4g32040* (KNOTTED 5) Gateway entry
MM 28 R	CTACGACTTCCCGGTCCGTTTACGT	
MM 29 F	CACCATGAAGCAATTCTGGAGTCCAAGTA	*At3g48330* Gateway entry
MM 30 R	TCAGTCCCCTCTCAGCTGGGCTTCA	
MM 31 F	CACCATGGCTCCGGGGCTTACTCAAACCG	*At2g47180* Gateway entry

MM 32 R	TCAAGCAGCGGACGGTGCGGTCACG	
MM 33 F	CCATGGGTATGTCGAAGATGGTTATGTTGCT	ASK13 Protein expression
MM 34 R	CTCGAGTTCAAAAGCCCATTGATTCTCCTTA	
MM 35 R	TAGGAGATGGACTTGTGGCTG	*ask13* (CS466214) homozygosity checking
MM 36 F	ACACGTCGTTTCTGATTCACC	
MM 37	ATATTGACCATCATACTCATTGC	T DNA Primer
MM 38 F	GAAGCTTATGTCGAAGATGGTTATGTTGC	*ASK13* Overexpression construct
MM 39 R	GTCTAGATCATTCAAAAGCCCATTGATTC	
MM 40 R	GCTCGAGTTCAAAAGCCCATTGATTCTCCTT	*ASK13 GFP* construct
MM 41 F	GAAGCTTATGTCGAAGATGGTTATGTTGC	
MM 42 F	CACCATGTCTGCGAAGAAGATTGTGTTGA	*ASK1* Gateway entry
MM 43 R	TCATTCAAAAGCCCATTGGTTCTCT	
MM 44 F	CACCATGTCGACGGTGAGAAAAATCACTC	*ASK2* Gateway entry
MM 45 R	TCATTCAAACGCCCACTGATTCTCA	
MM 46 F	CACCATGGCAGAAACGAAGAAGATGATCA	*ASK3* Gateway entry
MM 47 R	TCACTCGAACGCCCACCTGTTCTCA	
MM 48 F	CACCATGGCAGAAACGAAGAAGATGATCA	*ASK4* Gateway entry
MM 49 R	TCACTCGAACGCCCACTTGTTCTCA	
MM 50 F	CACCATGTCGACGAAGATCATGTTGAAGA	*ASK5* Gateway entry
MM 51 R	TCATTGAAAAGCCCATTGATTCTCC	
MM 52 F	CACCATGATGATAAAGGGTATGGCAGAAG	*ASK6* Gateway entry
MM 53 R	TCAGCGACAGTTTGAAAATGTGAGA	
MM 54 F	CACCATGTCGACAAAAAAGATCATGTTGA	*ASK7* Gateway entry
MM 55 R	TCATTCAAAAGCCCATTTATTGTCG	
MM 56 F	CACCATGTCGACGAAAAAGATCATGTTGA	*ASK8* Gateway entry
MM 57 R	TCATTCAAAAGCCCATTTATTCTCC	
MM 58 F	CACCATGTCGACGAAGAAGATCATATTGA	*ASK9* Gateway entry
MM 59 R	TCATTCAAAAGCCCATTTATTCTCC	
MM 60 F	CACCATGTCGACGAAGAAGATCATATTGA	*ASK10* Gateway entry
MM 61 R	TCATTCAAAACCCCATTGATTCTCC	
MM 62 F	CACCATGTCTTCGAAGATGATCGTGTTGA	*ASK11* Gateway entry
MM 63 R	TCATTCAAAAGCCCATTGATTCTCC	
MM 64 F	CACCATGTCTTCGAAGATGATCGTGTTGA	*ASK12* Gateway entry
MM 65 R	TCATTCAAAAGCCCATTGATTCTCC	
MM 66 F	CACCATGTCGAAGATGGTTATGTTGCTGA	*ASK13* Gateway entry

MM 67 R	TCATTCAAAAGCCCATTGATTCTCC	
MM 68 F	CACCATGTCTTCCAACAAGATTGTTTTGT	*ASK14* Gateway entry
MM 69 R	CTATTCAAAAGCCCATGCGTTTTCC	
MM 70 F	CACCATGTCTTCGAAAAAGATTGTGTTGA	*ASK19* Gateway entry
MM 71 R	CTAGGGTTTTGGAACTTGTTGTTTT	
MM72 F	CACCTCGTTTCTGATTCACCAACC	*ASK13 RNAi*
MM72 R	ACATCGTTCTTAAAAATTGCTCAA	
